# Global scale-free brain activity as a potential neural signature of visual information processing in aging

**DOI:** 10.3389/fnagi.2026.1770204

**Published:** 2026-04-01

**Authors:** Frigyes Samuel Racz, Zalan Kaposzta, Akos Czoch, Joshua T. Chang, Orestis Stylianou, Peter Mukli, Jared F. Benge, Andras Eke

**Affiliations:** 1 Department of Neurology, Dell Medical School, The University of Texas at Austin, Austin, TX, United States; 2 Mulva Clinic for the Neurosciences, Dell Medical School, The University of Texas at Austin, Austin, TX, United States; 3 Department of Physiology, Faculty of Medicine, Semmelweis University, Budapest, Hungary; 4 Oklahoma Center for Geroscience and Healthy Brain Aging, University of Oklahoma Health Sciences, Oklahoma City, OK, United States; 5 Vascular Cognitive Impairment and Neurodegeneration Program, Department of Neurosurgery, University of Oklahoma Health Sciences, Oklahoma City, OK, United States; 6 Oden Institute of Computational Engineering and Sciences, The University of Texas at Austin, Austin, TX, United States; 7 Department of Radiology and Biomedical Imaging, Yale University School of Medicine, New Haven, CT, United States

**Keywords:** 1/f, cognition, electroencephalography, aging, rapid visual information processing, scale-free activity, spectral slope

## Abstract

**Introduction:**

Confluent evidence suggests that aging is associated with a reduction in the spectral slope of neural recordings. This phenomenon has been hypothesized to reflect an increase in the ratio of excitatory and inhibitory (E/I) balance. As neural E/I ratio is considered essential for cognitive functioning, this study investigated whether spectral slope (*β*) predicts cognitive performance in the older adults and could serve as a potential neural signature of cognitive aging. Nevertheless, few studies have examined how spectral slope estimates obtained from both eyes-closed (EC) and eyes-open (EO) resting states relate to cognitive performance in the aging population.

**Methods:**

To address this gap, this study analyzed electroencephalography (EEG) recordings of 19 community-dwelling elderly adults (CDE, aged between 60 to 80 years) and 24 young control individuals (YC, aged between 21 to 32 years) collected during EC and EO states. Further, *β* was estimated from global power spectra after separating scale-free from oscillatory components in the power spectrum.

**Results:**

Reduced *β* was observed in CDE in contrast to YC in both physiological states. While the YC group indicated an increase in *β* when transitioning from EC to EO, this change could not be observed in CDE. Finally, a strong, anticorrelated relationship between β in EO and performance on a rapid visual information processing task, was typically seen only in the CDE group.

**Discussion:**

These results potentially indicate an altered E/I ratio in the CDE cohort, while the inverse relationship with task performance suggests that this pattern could reflect a compensatory brain mechanism supporting cognition during aging.

## 1 Introduction

Understanding the neural mechanisms of aging and the processes that drive age-related cognitive decline is a central goal of neuroscience (Harada et al., 2013). Even in normal aging, changes in attention, working memory, and processing speed begin to develop, while cognitive impairment–typically denoting cognitive abilities at least 1.5 standard deviations below that of one’s respective normative population (Abbott et al., 2019)–affects approximately 10 to 20% of adults aged 65 and older, with prevalence increasing exponentially with age (Petersen et al., 2018). Early detection is essential for interventions that may slow progression, improve quality of life, and facilitate planning such as financial management or lifestyle modifications. However, the underlying neural mechanisms of both normal and pathological aging remain incompletely understood (Fernández-Rubio et al., 2024). Electroencephalography (EEG) provides a non-invasive, cost-effective window into these mechanisms, enabling identification of alterations in neural dynamics that precede clinical symptoms and cognitive impairment (Rossini et al., 2007). In line, numerous electrophysiological markers have been proposed to capture how brain functioning alters in normal aging (Tanaka et al., 2024). Among these, scale-free ( 1/f -like) brain activity–characterized by a power-law decay in the power spectral density with increasing frequency–has emerged recently as a promising marker, with its spectral slope (*β*) likely reflecting underlying excitatory/inhibitory (E/I) balance (Voytek et al., 2015; Gao et al., 2017).

Specifically, converging evidence from independent studies indicates a flattening of β (i.e., a reduction in the magnitude of β) in healthy aging (Voytek et al., 2015; Dave et al., 2018; Pathania et al., 2022; Czoch et al., 2024). This flattening has been hypothesized to signify a shift toward increased excitation or reduced inhibition in cortical networks and is associated with behavioral outcomes (Waschke et al., 2021). Furthermore, the flattening of the power spectrum appears to be even more pronounced in mild cognitive impairment and Alzheimer’s disease (Martinez-Canada et al., 2023) compared to normal aging, potentially indicating its relevance to processes resulting in cognitive impairment and dementia. Nevertheless, while reduced *β* has been consistently observed in resting-state EEG–often limited to eyes-closed (EC) condition–its association with broader cognitive processes remains less clear. It has been shown that the reduction in β mediates age-related performance impairment in a visual working memory task (Voytek et al., 2015). Further, *β* predicted some features of evoked potentials in a lexical prediction task in both young and elderly cohorts; however, it did not mediate age-related decline in performance (Dave et al., 2018). In another work, while cognitive evaluation indicated reduced performance in aging for attention and delayed memory domains among others, the reduced 1/f slope only mediated performance in a coding subtest (Pathania et al., 2022). Recent evidence also points towards midlife *β*–in conjunction with oscillatory activity–may predict later-life decline in overall cognitive ability (Finley et al., 2024). However, the relationship between β and specific cognitive domains remains underexplored, particularly in eyes-open (EO) states, which engage visual processing and may reveal state-dependent adaptations in E/I balance (Barry et al., 2007). Indeed, recent analysis of the β indicates a reduction of E/I ratio when transitioning from EO to EC (Racz et al., 2024). On the other hand, previous studies have not systematically investigated how EO/EC variability in *β* is affected by healthy aging, and its potential relationship with performance on tasks sensitive to age-related cognitive decline, such as paired associates learning and spatial pattern recognition memory (Rabbitt and Lowe, 2000; Wild et al., 2008).

Building on these gaps, the present study analyzes EEG from a cohort of community-dwelling elderly (CDE) and young control (YC) individuals in both EC and EO resting states, estimating the global *β* from isolated scale-free spectra to avoid biasing effects introduced by the presence of oscillatory peaks. Global β estimates from both physiological states with performance outcomes from a set of cognitive tasks challenging various cognitive domains, focusing especially on tasks probing sustained attention and working memory. The results confirmed reduced β in aging compared to YC population but also uncovers a strong association with rapid visual information processing in the EO state only in the elderly group, suggesting compensatory mechanisms in aging.

## 2 Materials and methods

### 2.1 Participants and study protocol

A total of 25 YC and 22 CDE volunteers were recruited from students/employees of Semmelweis University and the general population of Budapest, Hungary. During the data screening process, 1 YC and 3 CDE participants were excluded from further analysis due to poor EEG data quality (see below), resulting in a final sample size of 24 YC and 19 CDE. Demographics information of the study cohorts is presented in Table 1, including mean age, women/men ratio, and level of education. The study was reviewed and approved by the Semmelweis University Regional and Institutional Committee of Science and Research Ethics (approval number: 2020/6) and was conducted in line with the Declaration of Helsinki. Inclusion criteria included age between 18 and 40 years for the YC, while age above 60 years for the CDE group, intact or corrected vision, and self-reported intact cognitive functioning with no subjective cognitive decline. Specifically, participants were screened through self-report to confirm preserved independence in daily activities, normal general cognition, absence of cognitive complaints, and no significant interference with work or social life. Participants were also instructed not to consume any caffeine at least 3 h before the measurement and have at least 6 h of sleep the preceding night. Exclusion criteria included diagnosis of mild cognitive impairment (MCI) or dementia, history of neuropsychiatric disorder (e.g., epilepsy, depression) or brain injury, current medication affecting cognitive performance, presence of any serious medical condition (e.g., pregnancy, severe cardiovascular pathology), or any condition preventing EEG measurement (e.g., metallic cranial implants). All participants were informed of study procedures and potential risks and provided written informed consent before measurements. Part of this dataset has been previously analyzed in Czoch et al. (2024) and Kaposzta et al. (2024). Screening relied mainly on participant self-report during recruitment and informed consent, without comprehensive neuropsychological evaluation.

**Table 1:** Demographic information of study groups.

**Variable**	**YC (*n* = 24)**	**CDE (*n* = 19)**	**Statistics**	***p*-value**
Sex	12F/12M	8F/11M	$\chi^{2}=0.2657$	p=0.6062
Age	24.5 [21, 32]	64 [60, 80]	z=−5.5725	$p<10^{-5}$
Level of education^1^	4 [3, 6]	6 [5, 6]	z=−4.5239	$p<10^{-5}$

For age and level of education, the median is shown with the minimum–maximum range in square brackets. YC: Healthy young; CDE: Community-dwelling elderly; F: Female; M: Male. ^1^Note that Level of Education is reported as a six-level ordinal variable according to how it is recorded during CANTAB assessment. Specifically, levels 1–2 denote leaving formal education before or at age 16, respectively, 3 denotes leaving formal education at 18/high school degree, and levels 4, 5, and 6 denote bachelor’s, master’s, and doctoral degree, respectively.

The study protocol was as follows. Participants were first informed of the study details and consent was obtained. This was followed by setting up the EEG system (~10 min) and the completion of three recording sections: (i) a resting-state EEG block involving EC and EO measurements (~7 min), (ii) a task-EEG block involving an n-back protocol (~27 min) (Kaposzta et al., 2020), a visual pattern recognition protocol (~10 min) (Stylianou et al., 2021), and a virtual spatial orientation task with simultaneous EEG collection (~10 min), and (iii) a concluding cognitive assessment block (~55 min), utilizing 7 + 1 tasks of the Cambridge Neuropsychological Test Automated Battery (CANTAB) without EEG acquisition (Robbins et al., 1994). The complete measurement protocol lasted about 2 h. In this study data collected in sections (i) and (iii) were only analyzed.

EEG was collected from 14 standard 10–10 scalp locations (AF3, AF4, F3, F4, F7, F8, FC5, FC6, T7, T8, P7, P8, O1, and O2) with an Emotiv EPOC+ wireless device (Emotiv Systems Inc., San Francisco, CA, USA) using Ag/AgCl electrodes with saline solution-soaked pads enabling scalp contact. CMS and DRL reference electrodes were positioned at P3 and P4 locations, respectively. Maximal contact quality was ensured as indicated by the EmotivPRO software. Brain activity was internally sampled by the headset at 2048 Hz, bandpass filtered between 0.2 and 45 Hz (5th order Sync filter) with additional notch filtering at 50 and 60 Hz, downsampled to 256 Hz, and then wirelessly transferred to a desktop computer performing data collection. The resting-state protocol involved 3–3 min of EC and EO data collection, respectively, while participants were sitting in a comfortable armchair in a quiet, dimly lit, electrically sealed room. During the EC condition participants were instructed to relax and keep their eyes closed, refrain from voluntary movements, avoid engaging in any structured mental activities, but remain awake. Once the 3 min had passed, participants were instructed to open their eyes, focus their gaze on a fixation cross presented to them as a computer display, and try to minimize blinking.

### 2.2 Cognitive testing

Participants completed a subset of cognitive tasks from the CANTAB inventory that are commonly utilized in studying cognitive aging (Csipo et al., 2019). All participants completed the cognitive assessment alone in a quiet room, using a 10.2-inch tablet computer. All tasks were presented in the participant’s native language and initiated with a training block to ensure task comprehension, which had to be passed before the participant could proceed to the live task assessment. In every case, the protocol started with a motor screening task (MOT) where the goal was to tap on a cross appearing on the screen at random locations, as fast as possible. The MOT task was utilized to ensure that potential between-group (YC vs. CDE) differences were not confounded by those in motor abilities. Then, the assessment continued with the following seven tasks: delayed match to sample (DMS), paired associates learning (PAL), immediate and delayed pattern recognition memory (PRM), reaction time (RTI), rapid visual processing (RVP), and spatial working memory (SWM). The standard output report from these assessment contains an abundance of variables (a total of 154) characterizing various aspects of performance, a majority of them encoding redundant/overlapping information (e.g., both mean and median of correct responses, number of correct and erroneous responses at a fixed trial count). To counter this and reduce the Type I error inflation, a total of 15 core output variables were selected (excluding MOT) for comparing performance between YC and CDE. The goal of this selection was to characterize primary cognitive domains (i.e., accuracy, speed, efficiency) probed by the given task while minimizing redundancy among performance metrics, and it was implemented based on previous literature to include CANTAB output metrics most commonly reported in similar studies focusing on cognitive aging (Siew et al., 2022; Cabral Soares et al., 2015; Abbott et al., 2019). Additionally, 10 output variables were also available as *z*-scores, that is, standardized to the normative population of the participant; these were then utilized to screen for clinically relevant cognitive impairment in the sample (see below). Short task descriptions with the targeted cognitive domains and the 15 selected output metrics are provided in Table 2, while a complete list and exploratory analysis of all available CANTAB output metrics are provided in the Supplementary Data Sheet 1.

**Table 2:** Cognitive tasks and selected output measures.

**Test**	**Description**	**Domain**	**Output measures**
MOT	Crosses appear on the screen sequentially in random locations. Task is to tap on each as fast as they can.	Sensorimotor skills	MOTML: mean latency from display of stimulus to response
DMS	Complex visual pattern is presented, then shown again with a delay (0, 4, and 12 s) among with three other, new patterns. Task is to identify and select the previously presented pattern.	Visual matching, short-term visual recognition memory	DMSPCAD*: Percentage of correct responses (all delays) DMSPEGC: Probability of error given the previous response was correct DMSPEGE* (*z*-score only): Probability of error given previous response was error
PAL	The participant opens boxes on screen, which contain unique patterns. After all boxes are opened, the patterns are presented one-by-one in randomized order. Task is to select the corresponding box to each pattern.	Visual memory and learning	PALFAMS28*: First attempt memory score (all conditions) PALTEA28*: Total errors (adjusted) in all conditions
PRM	Initially, complex visual patterns are presented sequentially. In the early recall task, pairs of patterns are presented immediately–one former and one new– also repeated in the late recall condition after ~20 min delay. Task is to select the familiar pattern.	Visual pattern recognition memory	PRMPCI*: Percentage of correct immediate responses (early recall task) PRMPCD*: Percentage of correct delayed responses (late recall task)
RTI	Participant holds finger in starting position at the bottom of screen, while one or five circles are indicated on the screen as possible target locations. Task is to tap on a yellow dot with minimal latency when it appears in one of the possible target locations.	Motor and mental response speed, impulsivity	RTIFES: Five-choice total error score RTIFMRT: Mean five-choice reaction time RTIFMMT: Mean five-choice movement time
RVP	Digits (2–9) are presented in a pseudorandom order at a 100/min rate. Participant is given a three-digit target sequence (or multiple ones on higher difficulty). Tasks is to respond with minimal latency when a target sequence is observed in the stream of digits.	Sustained attention and working memory	RVPA* (A prime): Subject’s sensitivity to target sequence RVPML: Mean response latency RVPPFA*: Probability of false alarm
SWM	Several closed boxes are shown on screen. The participant opens the boxes one-by-one in search of a yellow token. Boxes close after selecting them (maximum one box open at a time). Task is to find the yellow token without opening any of the boxes more than once. The number of boxes increase with difficulty (4–6–8–12)	Visuospatial memory, working memory, problem solving strategy	SWMBE468*: Between errors (opening a box where a token was already found) SWMTE468: Total errors (opening a box that was already opened) SWMS*: Search pattern strategy measure

For output measures, asterisk symbol indicates that the measure was also available in *z*-score format that is standardized to the participant’s demographic characteristics. MOT: Motor screening task; DMS: Delayed match to sample; PAL: Paired associates learning; PRM: Pattern recognition memory; RTI: Reaction time; RVP: Rapid visual processing; SWM: Spatial working memory.

### 2.3 EEG data selection and pre-processing

The initial and last 10 s of data from both EC and EO conditions were excluded to avoid transition effects. Then, raw EEG data were band-pass filtered using a 4th order, zero-phase Butterworth filter with cutoff frequencies 0.5 and 45 Hz (Kotowski et al., 2018; Racz et al., 2018b), and then visually inspected by two investigators independently to select continuous data segments free of overt artifacts (e.g., head movements, coughs). Overlapping segments (i.e., those deemed ‘clean’ by both investigators) were identified, and a continuous epoch of 72 s was isolated randomly for further analysis. This epoch length was chosen as the longest continuous time frame available for most participants in both physiological states. As a segment could not be identified in both EC and EO by this screening procedure in 1 YC and 3 CDE participants, they were excluded from further analysis.

Included data epochs were cleansed from artifacts using the independent component analysis (ICA) as implemented in the EEGLAB toolbox (Delorme and Makeig, 2004). Independent components were manually inspected by two investigators, and those likely associated with eye movements, blinks, and heart and skeletal muscle activity were removed. Then, 14-channel EEG was regained using a reverse ICA transformation, the data were standardized for each channel to be zero-centered with a unit standard deviation to account for potential differences in electrode impedances, and finally re-referenced to the common average electrode.

### 2.4 Spectral slope estimation and oscillatory power analysis

The continuous, 72-s EEG segments were divided into 9 non-overlapping epochs, each of a length 8 s. On these epochs, the power spectrum was decomposed into broadband scale-free and oscillatory components using the Irregular Resampling Auto-Spectral Analysis (IRASA) technique (Wen and Liu, 2016). This step is essential, as the presence of oscillatory peaks (e.g., the prominent alpha peak around ~10 Hz) can significantly bias *β* estimates, and thus it is preferred to obtain β from the isolated scale-free spectrum in which oscillatory peaks have been removed (He et al., 2010; Donoghue et al., 2022). IRASA performs this by exploiting the self-affinity property of scale-free (or *fractal*) signals (Mandelbrot and Van Ness, 1968), which does not hold for oscillatory (harmonic) signals. Further, resampling a fractal signal by factor h will only alter its distribution by a scaling factor of $h^{H}$ where H is the Hurst exponent and this property holds in the frequency domain as well (Mandelbrot and Van Ness, 1968). Assuming an additive model for brain activity composed of a broadband fractal and multiple oscillatory components, an algorithm can be devised to isolate the fractal component of the power spectrum using a pair of up- and down-sampled versions of the original signal (Yamamoto and Hughson, 1991, 1993). By using a broad set of non-integer factors h , this procedure can be carried out more robustly and with higher precision, yielding the IRASA technique (Wen and Liu, 2016). Once the raw power spectrum is decomposed, *β* can be estimated from the isolated fractal component without the biasing effects of oscillatory peaks. For theoretical background and details of the IRASA algorithm, please see the original article (Wen and Liu, 2016; Racz et al., 2022).

**Figure 1:**
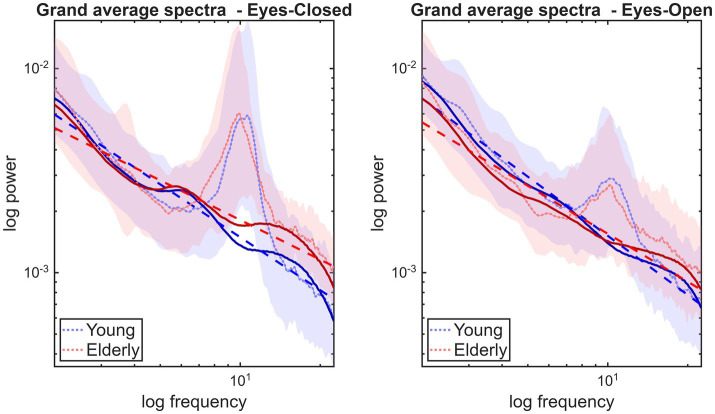
Separating broadband fractal and oscillatory components in the power spectrum. Dotted and thick lines denote the raw power spectrum and the isolated fractal component, respectively. The shaded areas indicate the standard deviation as observed in raw power spectra, while the dashed lines illustrate the power-law fit obtained from the fractal component.

To remain consistent with our previous analytical approach (Czoch et al., 2024), IRASA was employed using default parameter settings to isolate the fractal power spectrum in the 2–22.5 Hz frequency range. This range was set to avoid boundary filter effects that can be introduced by the up- and down-sampling procedures in IRASA (Racz et al., 2021). Fractal power spectra were obtained for each channel in each 8-s epoch, then averaged over channels and epochs to yield a robust global estimate before slope estimation. The β was then estimated using ordinary least squares regression of log-transformed fractal power on log-transformed frequency from the complete 2–22.5 Hz range. The group-average power spectra and the IRASA method are illustrated in Figure 1.

In addition to the spectral slope, isolated oscillatory alpha power – which has also been associated with inhibitory tone (Klimesch et al., 2007; Jensen and Mazaheri, 2010) and age-related cognitive decline (Babiloni et al., 2010) was also estimated. In that, raw (mixed) and isolated fractal power were first converted into dB, then oscillatory power was retrieved by subtracting the latter from the former. Band-limited power (BLP) in the alpha regime was obtained by averaging oscillatory power in the 8–13 Hz regime. Alpha BLP was computed for both EC and EO states, and alpha reactivity (Babiloni et al., 2010) was defined as the difference between EC and EO estimates.

### 2.5 Statistical analyses

Cognitive performance was contrasted between YC and CDE groups separately for the selected CANTAB output variables. A total of 15 performance measures were considered as described, as described previously. In all cases, normality of both variables was verified first using the Lilliefors test. Then, in cases of violation, a Mann–Whitney *U* test or a two-sample *t* test was performed. Outcomes were adjusted for multiple comparisons using the false discovery rate (FDR) method of Benjamini and Hochberg (1995) at α=0.05 . Available *z*-scored variables were utilized to screen for potential cognitive impairment. In cases where a higher value indicates poorer performance (e.g., PALTEA28), *z*-scores were multiplied by −1. The following performance threshold values were defined: *z*-score below −1.5 likely indicates clinically relevant cognitive impairment (CI) following (Abbott et al., 2019), while *z*-score those below −1.0 or −0.5 were defined as ordinal levels of subtle impairment (SI + and SI-, respectively) following Chipi et al. (2023) and Cabeça et al. (2018). The average of the 10 *z*-score metrics was also computed for each participant to provide a composite score of their overall cognitive performance relative to their demographic control group.

For spectral slopes, the study investigated the effect of age group (YC vs. CDE) and physiological state (EC vs. EO). First the normality of all variables was confirmed through the Lilliefors test. Then, a repeated-measures ANOVA with physiological state (EC vs. EO) as a within-group effect and age group as a grouping variable was performed. Sphericity was evaluated using the Mauchly’s test, and in cases of violation, the Greenhouse–Geisser correction was applied. In *post hoc* analyses, within- and between-group effects were assessed using the paired and independent two-sample *t* tests, respectively. Outcomes were adjusted for multiple comparisons using FDR at α=0.05 . The study also assessed if age was a continuous predictor of spectral slope. This was performed through the linear regression analysis with the model $B=B_{0}+B_{1}Age+B_{2}Group+B_{3}Age\times Group,$ where age in both groups (YC and CDE) was centered around the group mean to avoid collinearity between Age and Group . Furthermore, the same approach was utilized to assess if Level of Education (LOE) was predictive of *β*. These analyses were performed separately for EC and EO states. The same analytical framework was utilized to investigate between-group differences in alpha BLP.

Finally, this study explored whether there were any potential associations between spectral slopes and cognitive performance in the elderly population. This analysis only included those CANTAB output metrics that showed significant difference between YC and CDE. In these analyses, first the potential confounding effects of age, sex, and level of education were regressed out from both the EEG signature (β in EC and EO) and behavioral performance metrics. Then, the association between β and cognitive performance was assessed using the Pearson or Spearman correlation, depending on data normality.

### 2.6 Channel-wise analyses

To explore whether neural signatures show topological specificity, the previously described analyses were also executed on the channel level. Specifically, spectral slopes and oscillatory alpha activity were obtained for every channel instead of only from grand-averaged spectra. The outcomes were then evaluated using a repeated-measures ANOVA model with age group as the grouping variable, and physiological state and scalp location as within-group effects. *Post hoc* comparisons and correlation analyses between β and CANTAB metrics were executed according to the same principles as described for global estimates of β.

### 2.7 Validation and sensitivity analyses

While this study opted to utilize IRASA for separating fractal and oscillatory power spectra and estimate β, we considered it important to (i) verify our outcomes using a different, data-driven approach and (ii) explore whether power spectra express significant bimodality [i.e., multiple scaling regimes with different slopes, as observed previously in Miller et al. (2009) and He et al. (2010)] in the considered frequency range. Arguably, the most popular technique to characterize the fractal (or also called *aperiodic*) component of neural activity is the spectral parametrization (or ‘fitting oscillations & one over f’, FOOOF) method (Donoghue et al., 2020); however, FOOOF is limited in its capability to assess spectral multimodality. On the other hand, this study has recently introduced an analysis tool termed as the multi-modal spectral parametrization method (MMSPM; Racz et al., 2025). MMSPM utilizes the FOOOF workflow to separate oscillatory and aperiodic components, but instead of a Lorentzian, it assumes a piecewise, constrained power-law model, thus allowing for both concave- and convex-type bimodality. Notably, in cases of unimodal (i.e., single scaling regime) assessment, MMSPM is equivalent to FOOOF with fixed ‘knee’ parameter. Therefore, utilizing MMSPM could allow us to verify the outcomes of the IRASA analysis and explore possible spectral bimodality in a statistically rigorous manner. Further, this study re-analyzed the dataset with the same analytical pipeline but using MMSPM instead of IRASA. MMSPM was utilized with unimodal settings for confirmatory purposes, while with adaptive bimodal settings to explore potential multimodality. Further details of this analysis are provided in the Supplementary Data Sheet 1.

Furthermore, as screening for cognitive impairment was primarily based on self-report, this study could not completely exclude the possibility that some of the participants in the CDE cohort were already developing MCI. Therefore, two additional analyses were performed to confirm that low-performing participants–who potentially had cognitive impairment–did not significantly bias study outcomes. First, the study excluded participants from the elderly cohort who had z<−1.5 in any of the PAL and SWM metrics, as these tasks were shown by previous literature to be sensitive in identifying cognitive impairment (Égerházi et al., 2007; Sabahi et al., 2022). Then, all analyses on this reduced dataset were repeated. Then, subgroup analysis was performed, where the sorted participants of the (full) elderly cohort into ‘potential impairment (PI) and ‘probably healthy’ (PH) subgroups based on whether they had performance measures in *any of the tasks* with z<−1.5 or not, respectively. Then, all correlation analyses were replicated in a linear regression framework using the model $C=B_{0}+B_{1}\beta \times SG$ , where C and SG denote the CANTAB performance measure and the subgroup label, respectively. A significant β×SG interaction in this model would indicate that a potential β~C relationship is affected by the subgroup that is whether elderly participants are categorized as PH or PI.

## 3 Results

### 3.1 Age-related difference in cognitive performance

Results regarding differences in raw performance measures between HY and HE have been reported previously (Czoch et al., 2024; Kaposzta et al., 2024); for completeness, a brief summary is provided in Table 3. Performance in the MOT task was comparable among the two groups, supporting that performance differences in the other tasks were unlikely to be confounded by impaired motor function in any of the groups. It took significantly longer time for the elderly cohort to complete the cognitive assessment block (HY: 53.37 min vs. HE: 57.92 min, Mann–Whitney *U* test, $p<10^{-5}$ ). Out of the 15 screened, total of 8 performance measures were found significantly different between HY and HE, and without exception these indicated lower performance in the elderly cohort (Table 3). These results mainly indicated reduced task performance in the PAL, RVP, and SWM tasks, while increased response latency in the RTI and RVP tasks. A complete report of all 154 CANTAB output measures is provided in Supplementary Data Sheet 2 (with additional description in the Supplementary Data Sheet 1); this exploratory analysis reported significant between-group differences in a total of 54 metrics, indicating reduced performance in the elderly compared to the young cohort in all cases.

**Table 3:** Contrasting CANTAB output measures between young and elderly groups.

**Measure**	**Young**	**Elderly**	**Statistic**	***P*-value**	**ES**
DMSPCAD	87.00 [83.50; 93.00]	87.00 [80.00; 87.00]	0.9724	0.3308	*r* = 0.15
DMSPEGC	0.11 [0.06; 0.12]	0.12 [0.11; 0.12]	−1.2664	0.2370	*r* = −0.19
PALFAMS28	16.54 (2.60)	11.95 (3.15)	*t_41_* = 5.2348	***p* < 10** ^**−4**^	*d* = 1.58
PALTEA28	5.00 (4.27)	13.16 (7.80)	*t_41_* = −4.3710	**0.0004**	*d* = 1.32
PRMPCI	94.44 [86.11; 100.00]	88.89 [83.33; 94.44]	2.0396	0.0690	*r* = 0.31
PRMPCD	94.44 [83.33; 100.00]	88.89 [83.33; 94.44]	1.4002	0.2018	*r* = 0.21
RTIFES	0.00 [0.00; 1.00]	0.00 [0.00; 0.00]	1.8482	0.0881	*r* = 0.28
RTIFMRT	341.51 (39.81)	391.45 (38.63)	*t_41_* = −4.1390	**0.0006**	*d* = 1.25
RTIFMMT	216.26 [181.90; 235.58]	245.90 [202.40; 294.91]	−2.0176	0.0654	*r* = −0.31
RVPA	0.94 [0.91; 0.97]	0.90 [0.88; 0.93]	*z* = 3.0451	**0.0058**	*r* = 0.46
RVPML	466.62 [406.32; 497.80]	568.00 [506.97; 630.25]	*z* = −4.2675	**0.0001**	*r* = 0.65
RVPPFA	0.00 [0.00; 0.01]	0.00 [0.00; 0.01]	−1.0944	0.2933	*r* = −0.17
SWMBE468	5.00 [0.00; 11.00]	19.00 [5.00; 23.00]	*z* = −2.9072	**0.0078**	*r* = 0.44
SWMTE468	6.38 (6.48)	15.79 (10.81)	*t_41_* = −3.5437	**0.0030**	*d* = 1.07
SWMS	6.71 (2.80)	8.95 (2.48)	*t_41_* = −2.7326	**0.0173**	*d* = 0.82

Group expected values are either presented as mean (standard deviation) or median (inter-quartile range) format, depending on data normality. ES denotes the effect size, captured as Cohen’s *d* or rank-biserial correlation *r*, depending on data normality. Measures that indicated significant between-group differences in performance are denoted in bold.

**Figure 2:**
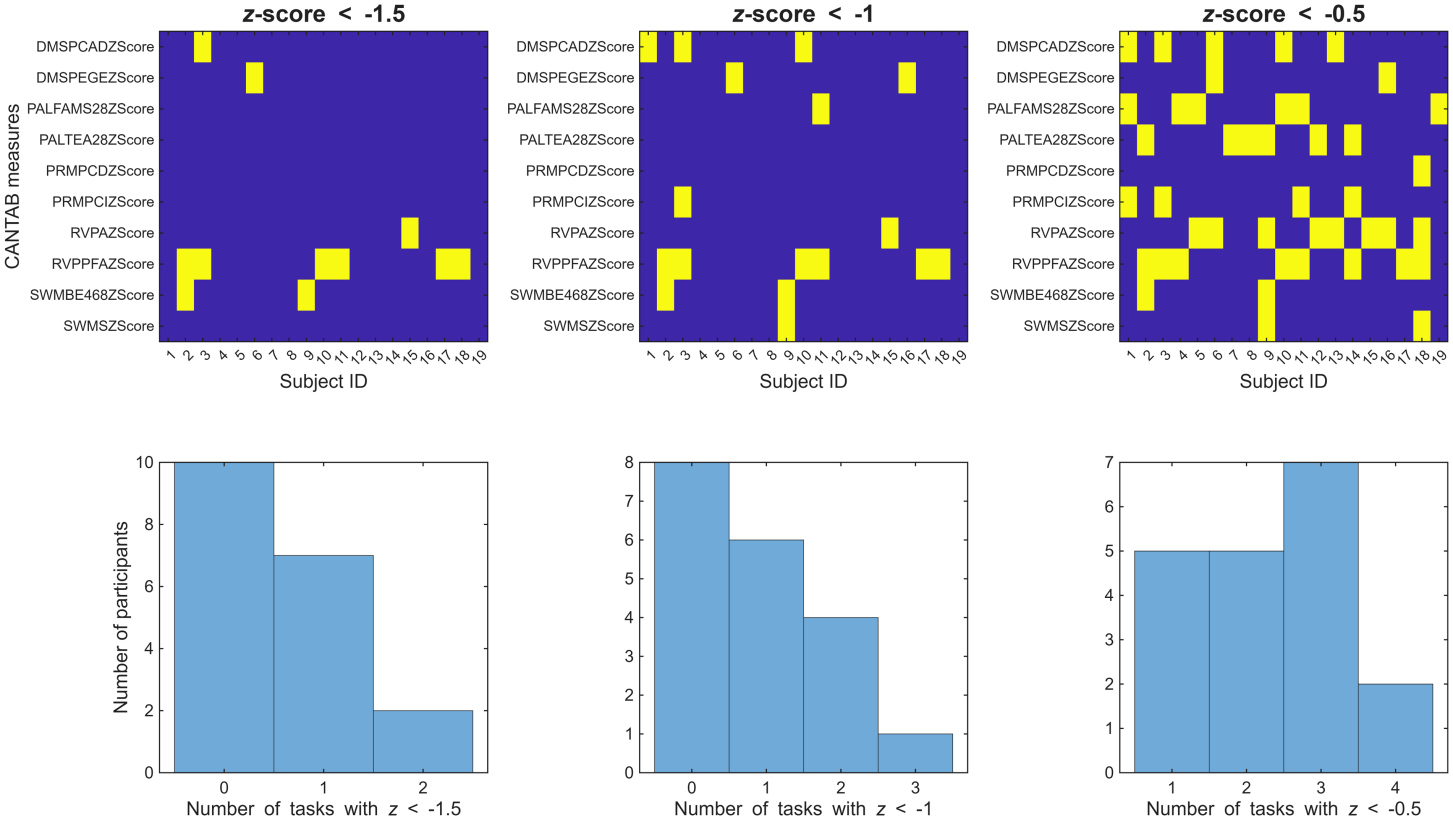
Upper panels indicate cases (yellow cells) where task performance of the given subject was below the pre-defined thresholds (from left to right: −1.5, −1.0, and −0.5). Lower panels show the corresponding distribution (histogram) of participants with *x* number of task performances below the given threshold.

In order to ensure the older adults in the sample were not experiencing substantial cognitive decline suggestive of MCI or dementia, age-adjusted *z*-scores provided in the CANTAB report were examined; outcomes of this analysis are presented in Figure 2. A total of 2, 7, and 2 participants had *z*-scores below −1.5 for the DMS, RVP, and SWM tasks, while all participants performed above *z* = −1.5 in all other tasks. The group average of total composite (i.e., average over all tasks) *z*-score was 0.1599 ± 0.2567, indicating performance better than the normative population ( p=0.0142 , $t_{18}=2.7149$ , and d=0.5965 , one-sample t test) on the group level. The lowest global (average) *z*-score value in the sample was *z* = −0.2370. Left and middle panels of Figure 2 show only sporadic cases with *z* < −1.5 and *z* < −1.0, respectively (except for the RVPPFA measure), while the rightmost panels show that multiple participants had task performances with *z* < −0.5 (but *z* > −1.0), although none of them had poor relative performance in more than four tasks. As similar performance variability in multiple cognitive domains is commonly observed in normative studies on cognitive aging (Binder et al., 2009) all participants were retained from primary analyses, the potential effect of low-performing participants was assessed in the subsequent validation analyses (Figure 2).

### 3.2 Effect of age and physiological state on global spectral slope

**Figure 3:**
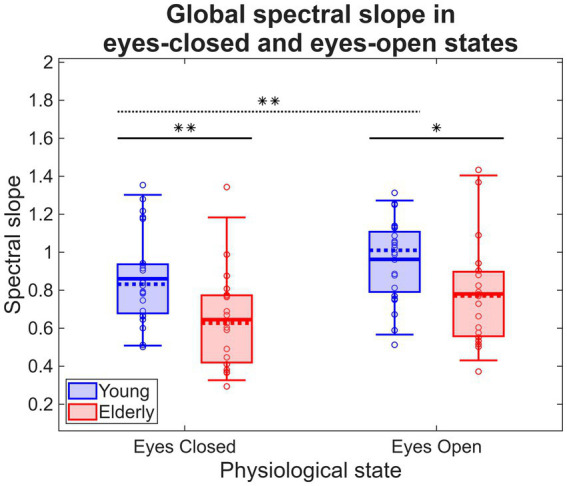
EEG spectral slopes in young and elderly cohorts. Thick horizontal lines indicate significant between-group differences, while dotted line denotes significant within-group difference. * and ** indicate *p* < 0.05 and *p* < 0.01, respectively.

The repeated-measures ANOVA indicated a significant main effect of group ( F=9.2337 , p=0.0041 , and $\eta_{p}^{2}=0.1838$ ) and physiological state ( F=10.076 , p=0.0029 , and $\eta_{p}^{2}=0.1973$ ), however no significant age group × physiological state interaction was found ( F=0.1972 , p=0.6593 , and $\eta_{p}^{2}=0.0048$ ). *Post hoc* between-group analysis revealed decreased *β* in CDE compared to YC on both EC (YC: 0.8604±0.2349 vs. CDE: 0.6453±0.2586 , $t_{41}=2.8525$ , p=0.0068 , and d=0.8598 ) and EO (YC: 0.9624±0.2101 vs. CDE: 0.7805±0.2826 , $t_{41}=2.4221$ , p=0.0199 , and d=0.7301 ) states, while *post hoc* within-group analysis revealed a significant increase in β for YC ( $t_{23}=2.9613$ and p=0.0070 , and d=0.5845 ) but not in the CDE group, as for the latter the difference was marginally significant ( $t_{18}=1.8776$ , p=0.0767 , and d=0.4125 ). These results are illustrated in Figure 3. When investigating age as a continuous predictor of *β*, the linear regression analysis indicated the previously observed significant main effect of Group in both EC and EO conditions. However, neither the main effect of Age nor the Age×Group interaction reached significance in either physiological states, indicating no continuous relationship between age and *β*. Similar outcomes were obtained when investigating the relationship between level of education (LOE) and β, although the main effect of LOE was marginally significant in EC ( $B_{1}=-0.1124$ , p=0.0693 , $R^{2}=0.2877$ ), indicating an inverse trend between education level and spectral slope.

### 3.3 Association between spectral slope and cognitive performance

**Figure 4:**
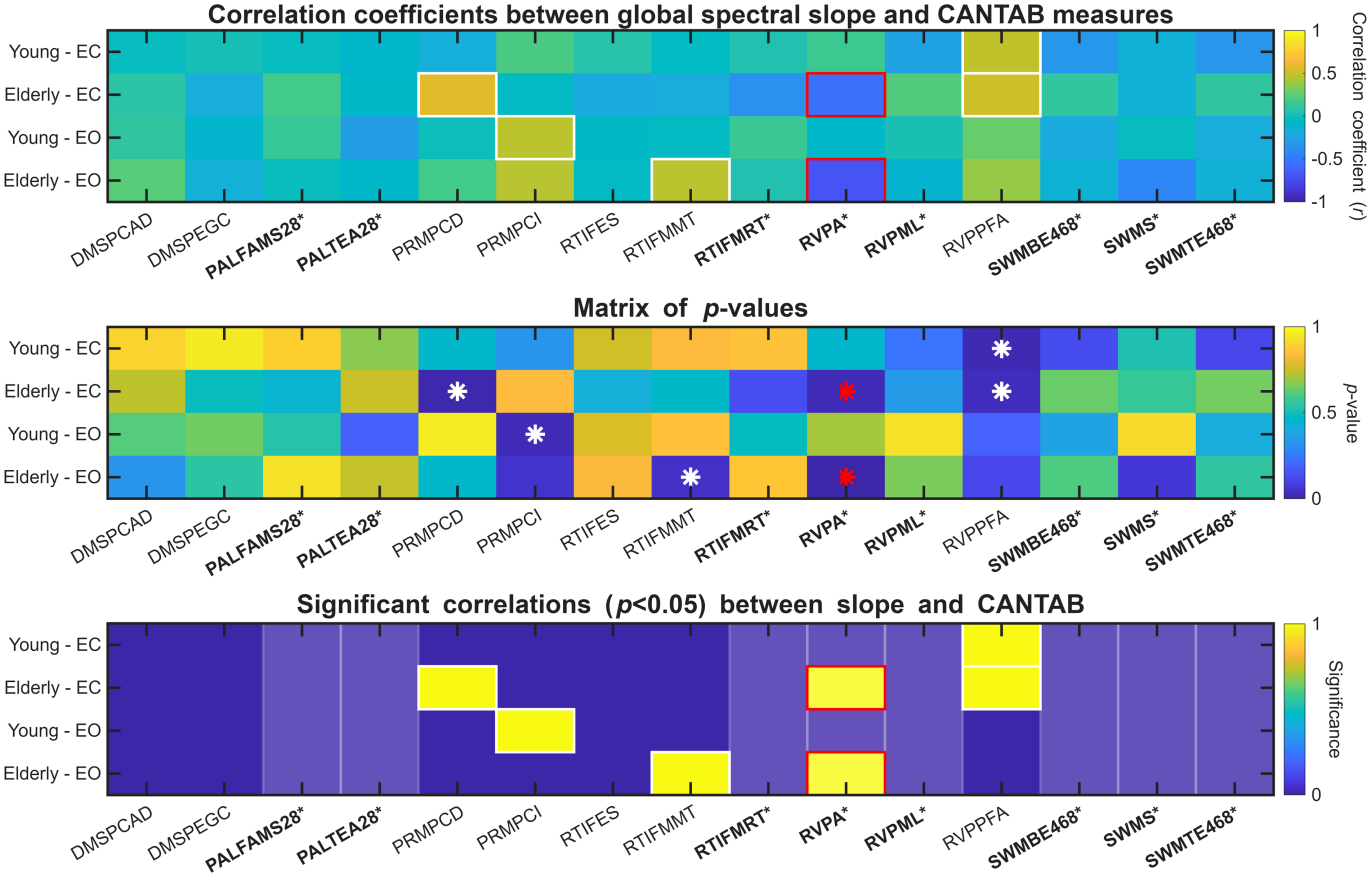
Complete correlation analysis between spectral slope *β* and CANTAB output measures. The top matrix shows the Pearson- or Spearman correlation coefficient (*r*) values depending on data normality, while the middle and bottom panels present the corresponding *p*-values and indicated statistical significance (*p* < 0.05), respectively. In all panels, CANTAB measures that were significantly different between young and elderly groups are denoted in bold and marked with an asterisk. This is also further illustrated in the bottom panel by slightly lighter shading. β-CANTAB correlations that were statistically significant (*p* < 0.05) are indicated by thick frames in the top and bottom panels, while asterisk symbols in the middle panel. Finally, on all panels, significant correlations for CANTAB measures that indicated a performance difference between young and elderly cohorts are denoted with red color, while the rest is marked in white.

A complete correlation analysis–involving all fifteen CANTAB measures and all four age group-physiological state combinations – is illustrated in Figure 4, with details provided in Supplementary Data Sheet 3. This analysis indicated a significant relationship between β and CANTAB performance measures in a total of seven cases, involving the PRM, RTI, and RVP tasks and both age groups and physiological states. However, among those cognitive measures that indicated a significant difference in performance between YC and CDE, only RVP expressed a significant relationship with *β*, and this effect was observed exclusively in the elderly cohort (see Figure 4, bottom panel).

**Figure 5:**
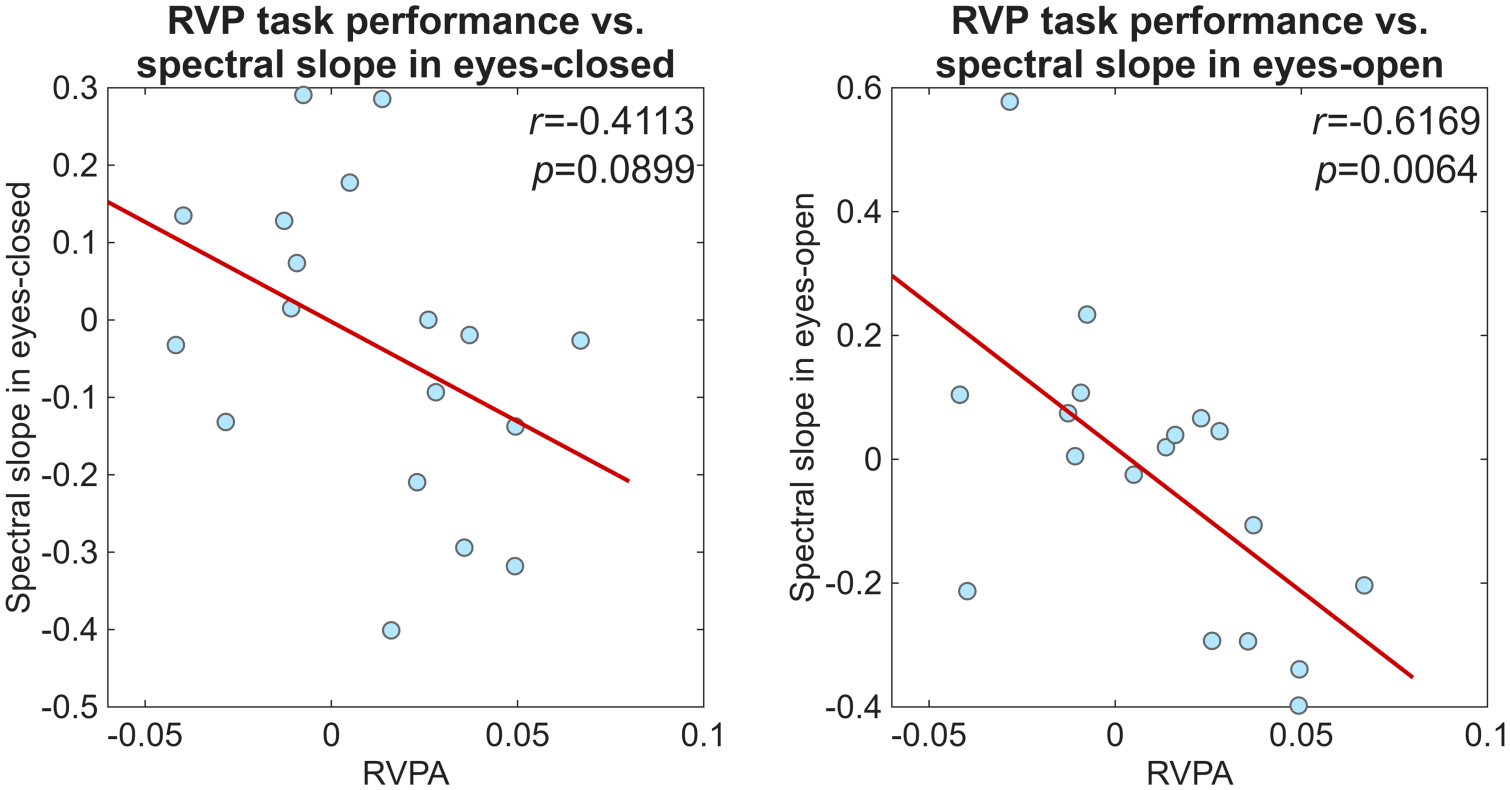
Correlation between RVPA and EEG spectral slope in eyes-closed (left) and eyes-open (right) conditions in the community-dwelling elderly group. The figure shows analysis of data after one outlier has been removed, whose RVPA score was 3.5 standard deviation below the group mean. The thick red line indicates the least-squares fit.

Specifically, the Spearman correlation coefficient for the RVPA–β relationship was significant both in the EO (*r* = −0.7000, *p* = 0.0012) and EC (*r* = −0.5351, *p* = 0.0198). Inspection of the data indicated an outlier subject with RVPA score below the group mean with 3.5 standard deviations after removing confounding variables (see Methods). Excluding this participant rendered the data normally distributed with Pearson correlation analysis indicating the relationship with *β* significant in the EO state (*r* = −0.6169, *p* = 0.0064), while marginally significant in EC (*r* = −0.4113, *p* = 0.0899). The RVPA–β relationship is further illustrated on Figure 5 below, while that on the complete sample (without excluding the outlier) is shown on Supplementary Figure 1. The RVPA–*β* relationship was anticorrelated in both EC and EO, indicating that lower *β* values were associated with higher RVPA scores.

### 3.4 Oscillatory alpha activity

**Figure 6:**
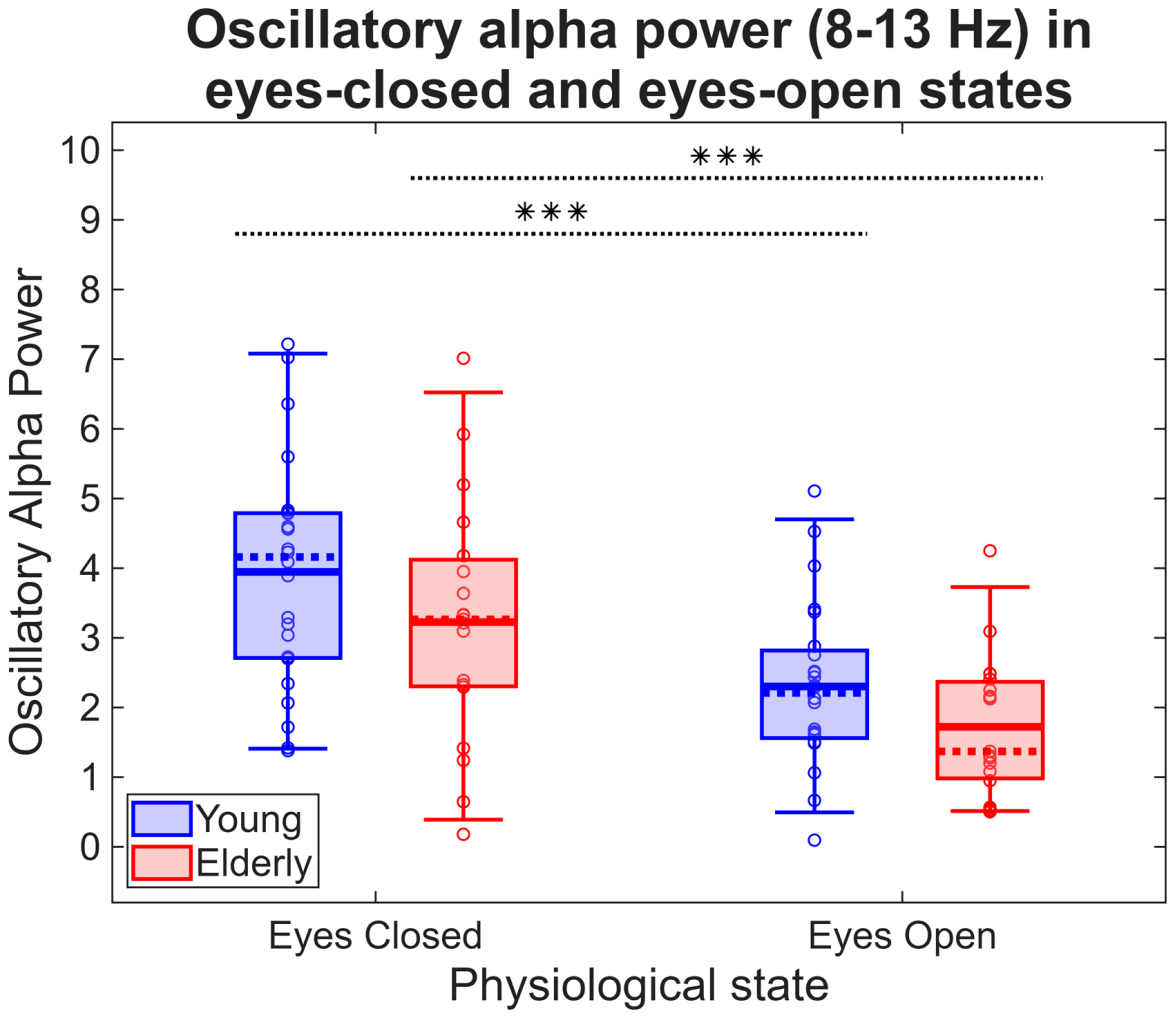
Oscillatory alpha BLP in young and elderly cohorts. Dotted horizontal lines denote significant within-group difference in response to change in physiological state. *** indicates *p* < 0.0001.

Repeated-measures ANOVA indicated a strong main effect of physiological state ( F=67.303 , $p<10^{-9}$ and $\eta_{p}^{2}=0.6214$ ) but no main effect of age group ( F=2.7448 , p=0.1052 , and $\eta_{p}^{2}=0.0627$ ). The age group × physiological state interaction was not significant ( F=0.1365 , p=0.7137 , and $\eta_{p}^{2}=0.0033$ ). *Post hoc* analysis revealed that while a significant reduction in oscillatory alpha BLP was found in both YC ( ΔBLP=−1.65±1.23 , $t_{23}=6.5413$ , $p<10^{-5}$ , d=1.2911 , one-sample *t-*test) and CDE ( ΔBLP=−1.50±1.27 , $t_{18}=5.1516$ , $p<10^{-4}$ , d=1.1318 , one-sample *t* test) cohorts when transitioning from EC to EO. However, there was no between-group difference in the extent of this change (i.e., alpha reactivity, $t_{41}=0.3695$ , p=0.7137 , d=0.1114 , two-sample *t* test). Oscillatory alpha BLP results are illustrated on Figure 6.

### 3.5 Replication of analyses on the channel level

**Figure 7:**
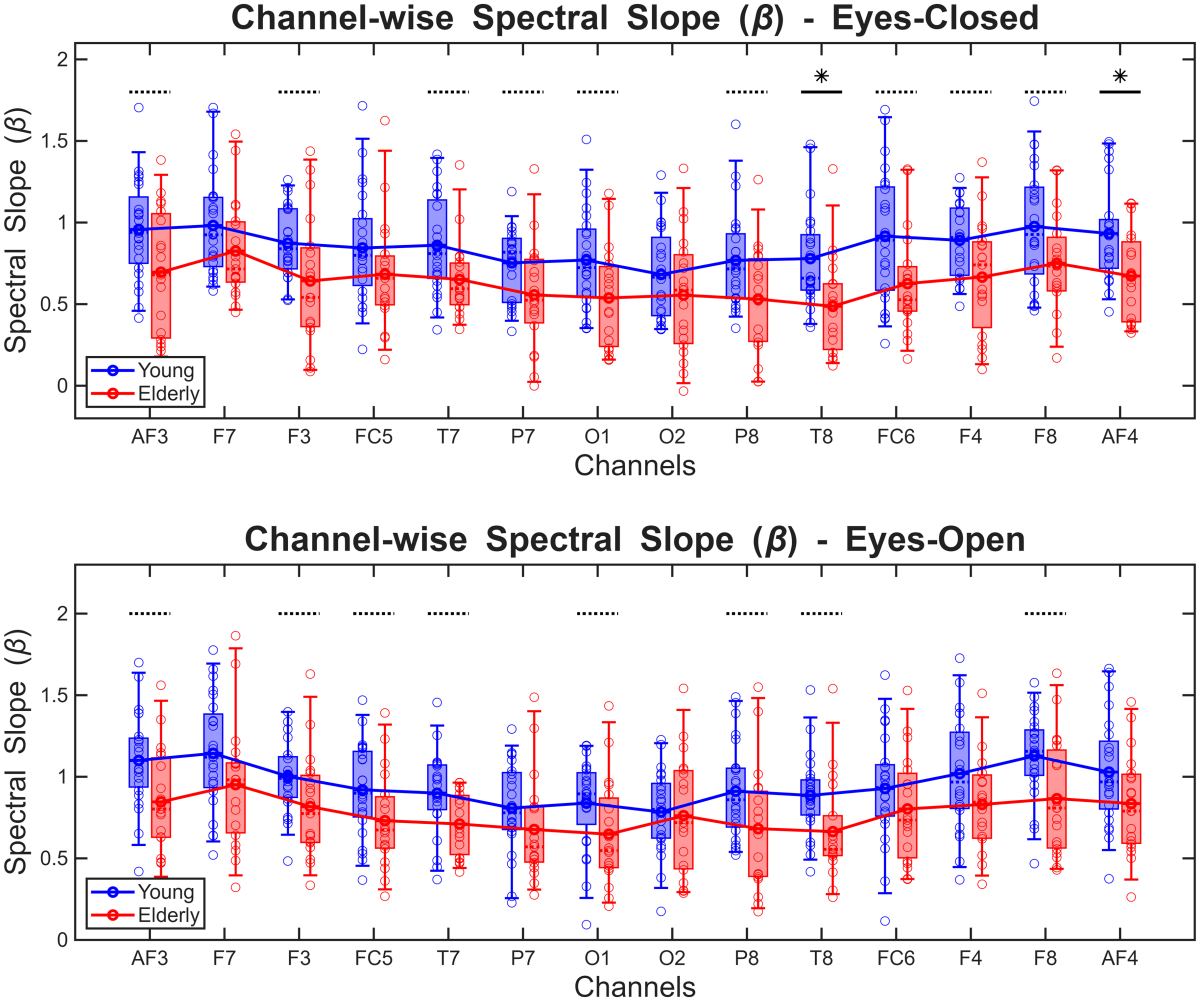
Channel-wise EEG spectral slopes in young and elderly cohorts, in eyes-closed (upper) and eyes-closed (lower) states. Thick horizontal lines indicate significant between-group differences, while dotted lines denote between-group differences rendered non-significant after multiple comparisons adjustment. * indicates *p* < 0.05.

When considering channel-wise estimates of *β*, the repeated-measures ANOVA model indicated a significant main effect of age group ( F=10.057 , p=0.0029 , $\eta_{p}^{2}=0.1970$ ), physiological state ( F=10.829 , p=0.0021 , $\eta_{p}^{2}=0.2089$ ), and scalp location ( F=13.387 , $p<10^{-9}$ , $\eta_{p}^{2}=0.2461$ ). However, none of the interaction terms–including the age group × physiological state × scalp location were statistically significant ( p>0.35 in all cases). Between-group differences are shown on Figure 7, while the same data is also shown on Supplementary Figure 2 to illustrate changes related to physiological state. Overall, these outcomes confirm the patterns observed in the global spectral slope, with *β* generally lower in the CDE compared to the YC group, and a stronger physiological state-related response in the YC group.

**Figure 8:**
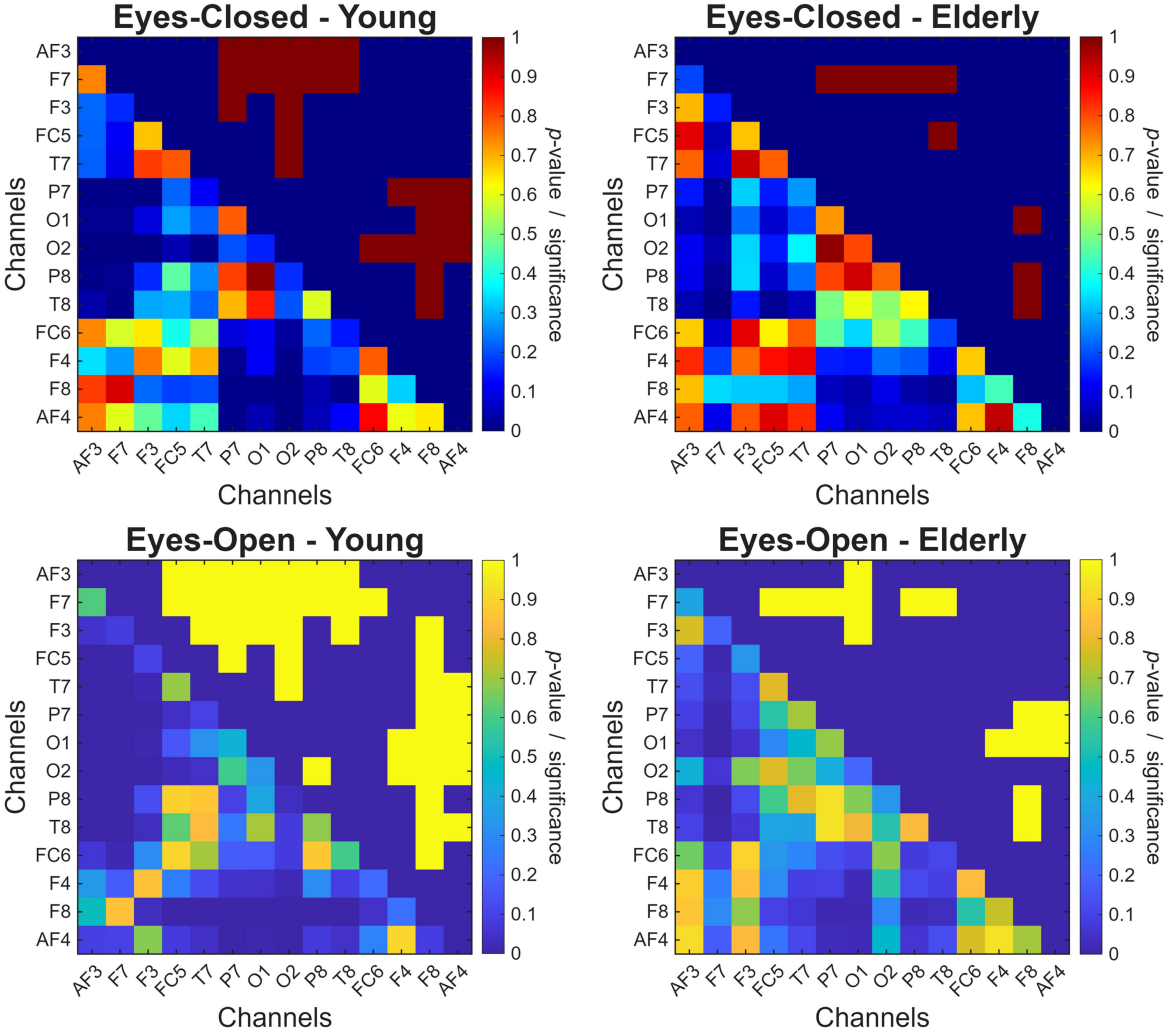
Within-group comparisons of channel-wise β estimates. In each panel, the lower triangular shows *p*-values from contrasting β across all channel pairs, while the upper triangular indicates the statistical significance (1 denoting *p* < 0.05 after multiple comparisons adjustment). The top panels show data from eyes-closed condition in the young (left) and elderly (right) cohorts, while the bottom panels depict the same for the eyes-open condition.

Since the ANOVA analysis indicated strong main effect of scalp location but no interactions with age group or physiological state, *β* estimates were compared between different channels for each four age group–physiological state combination separately (Figure 8). From the comparison matrices and the data presented on Figure 7 it is apparent that β is generally lower over posterior electrodes (i.e., P7, P8, O1, and O2) compared to frontal scalp locations, and this pattern is expressed more pronounced in the YC compared to the CDE group, where most significant differences (compared to other channels) were attributed to F7 and F8 locations.

Finally, channel-wise β estimates were also evaluated for correlations with CANTAB performance metrics; these outcomes are summarized in Supplementary Figure 3 and reported in detail in Supplementary Data Sheet 4. Overall, this pattern aligns with those observed in the global spectral analysis (Figure 4). Notably, the significant RVPA–*β* correlation observed in CDE (Figure 5) is present over 6 out of 14 channels in EC and 13 out of 14 channels in EO. Similarly broad association patterns were observed in other cases (e.g., PRMPCD-*β* for HE-EC, PRMPCI-β for YC-EO, and CDE-EO), indicating that global β efficiently captured ongoing activity pattern with relatively low information loss (i.e., significant correlations with CANTAB measures were not primarily driven by a single location).

In summary, channel-wise analyses further confirmed a widespread decrease in β across the cortex, with the CDE group exhibiting a close-to-uniform spatial distribution of β. Significant correlations with CANTAB performance metrics were observed broadly across the cortex instead of being concentrated at specific scalp locations.

### 3.6 Validation and sensitivity analyses

Outcomes of the MMSPM analysis are detailed in the Supplementary Data Sheet 1. Bimodality could not be reliably confirmed with MMSPM on the individual level (statistically significant spectral bimodality was only exhibited by less than 50% of participants in each study group, see Supplementary Table 1); therefore, we proceeded with unimodal estimation of *β* (Supplementary Figure 4). This analysis yielded results almost identical to those obtained via IRASA, both with regards to between-group differences in the spectral slope β (Supplementary Figure 6) and its correlation with cognitive performance (Supplementary Figure 7).

As shown in Figure 2, only 2 out of 19 elderly participants had *z*-scores below −1.5 for the SWM task (none for the PAL task), while a total of 9 out of 19 participants had a performance below the z=−1.5 threshold in at least one measure, which might indicate – but not confirm – cognitive impairment. Repeating the complete analysis pipeline after excluding participants CDE#2 and CDE#9 yielded identical results to those on the complete sample; these results are presented on Supplementary Figures 8–10. In the subgroup analysis, two cases were found in total where the interaction term was significant: when investigating the relationship between *β*-EC and PRMPCD ( $B_{1}=35.37$ , SE=15.76 , $t_{15}=2.2435$ , p=0.0404 , and $R^{2}=0.519$ ) and between β-EO and PRMPCI ( $B_{1}=34.92$ , SE=15.45 , $t_{15}=2.2612$ , p=0.0390 , and $R^{2}=0.418$ ). These cases are illustrated in Supplementary Figure 11. Note that while these cases indicated a difference in the *β* ~ CANTAB relationship depending on subgroup association, the involved CANTAB performance metrics (PRMPCD and PRMPCI) showed comparable performance between the YC and CDE groups (Table 3). In summary, these additional analyses demonstrate that, although the presence of individuals with potential cognitive impairment in the CDE cohort cannot be excluded with absolute certainty, this likely had minimal effect on the reported findings.

## 4 Discussion

This study is an extension of our previous works (Czoch et al., 2024; Kaposzta et al., 2024), by including EO in addition to EC state, and aiming to (i) investigate how change in physiological state alters global EEG spectral slope, (ii) compare *β* between younger and older adults, and (iii) explore how β in both of these two states may be associated with cognitive performance.

### 4.1 Resting-state EEG patterns in cognitive aging

Analyzing resting-state EEG has both advantages and disadvantages; the method is a simple yet effective and valuable means to scope baseline brain activity, but it remains challenging to elucidate the exact neural mechanisms for patterns observed in resting-state. Introducing the trivial physiological ‘intervention’ of transitioning from EC to EO can ameliorate this issue to some extent, although most of the EEG literature focuses only on EC activity–as an example, only a small minority of resting-state EEG markers in psychiatric disorders are assessed in EO (Newson and Thiagarajan, 2019). This is likely due to the lack of certain artifacts in EC data such as blinks or eye movements. On the other hand, EC and EO resting-state represents substantially different modes of brain function (Barry et al., 2007), with the latter not only characterized by increased arousal but also visual information processing. Besides the hypothesis that *β* of local field potential recordings is corresponding to the E/I ratio (Gao et al., 2017), resting alpha power was also previously associated with inhibitory activity (Klimesch et al., 2007; Jensen and Mazaheri, 2010). Consequently, alpha reactivity–defined as reduction in occipital alpha power when opening the eyes (Babiloni et al., 2010)–may reflect changes in the E/I ratio during the transition from EC to EO (Racz et al., 2024). While the spectral slope *β* showed an association with RVP task performance, we also wanted to explore if resting alpha power carries the same information in terms of being a neurophysiological indicator of cognitive performance.

While alpha BLP significantly decreased in both study groups when transitioning from EC to EO, the extent of this change was comparable and no between-group differences were observed. This is in contrast with results regarding the spectral slope, where *β* was reduced in HE compared to HY in both EC and EO states, and physiological state had a significant main effect. Furthermore, while the agegroup×physiologicalstate interaction effect was not significant, a statistically significant increase in β could only be identified in the young but not in the elderly group (see Figure 3). Furthermore, global alpha reactivity showed no association to any cognitive performance measures when evaluated the same way as for spectral slope, in contrast to the observed *β* ~ RVPA association. Based on the outcomes regarding β we hypothesize a reduced ability in the elderly cohort to adjust cortical E/I ratio in response to opening the eyes that might be associated with sustained attention (as probed by the RVP task), although this is not confirmed by resting alpha BLP and requires further investigation. It must be noted, however, that our electrode montage only contained two electrodes over the occipital cortex (O1 and O2) where resting alpha rhythms are most prevalent (Barry et al., 2007), while most of our signal was collected from frontal and prefrontal scalp regions, likely impacting our analysis outcomes.

### 4.2 Physiological state-related changes in spectral slope

It is important to note that while EO can be reasonably hypothesized as a state of decreased inhibition compared to EC – as also indicated by reduced alpha power (Jensen and Mazaheri, 2010), *β* increased in the CDE cohort when transitioning from EC to EO indicating an increase in inhibition (or reduction in excitation), counterintuitive to the E/I ratio hypothesis (Gao et al., 2017). This is likely to do with our range of estimating *β* (i.e., from 2–22.5 Hz). While the E/I ratio hypothesis of Gao and colleagues – originally posited for the 30–50 Hz regime–has been confirmed by numerous studies. Further, reports also indicate that the relationship between E/I ratio and the *β* may be reversed in the low-frequency (i.e., <5 Hz) range (Becker et al., 2018; Muthukumaraswamy and Liley, 2018). Furthermore, recent evidence (from a YC population) also suggests that EC-to-EO transition can evoke a simultaneous increase in *β* in the low-frequency regime (<5 Hz), and reduction in the high-frequency regime (>20 Hz) (Racz et al., 2024). Therefore, care must be taken when interpreting an increase in β as a decrease in E/I ratio on the new sample, without having simultaneous slope estimates from the 30–50 Hz regime, too. Unfortunately, inherent characteristics of our dataset (such as built-in filtering in the EEG system) did not allow us to incorporate a broader frequency regime for slope estimation (Supplementary material in Racz et al., 2021); however, it is our immediate goal to address this issue in the future. Considering the decrease in alpha BLP in EO, it appears more likely that our slope estimates were primarily driven by low-frequency activity and thus indicate the phenomenon observed by Becker et al. (2018), Muthukumaraswamy and Liley (2018), and Racz et al. (2024), and thus an increase in *β* indeed reflects an increase in E/I ratio.

### 4.3 Scale-free brain activity and cognitive performance

In an earlier study (Czoch et al., 2024), focusing only on EC activity, a similar anticorrelated relationship between β and RVPA was observed over right-frontal scalp locations. However, that pipeline did not account for potential confounding factors, and those results that might have been affected by age, sex, and level of education. The present analysis–controlling for these variables and excluding one outlier participant–identified this association as significant in EO (*p* = 0.0064) but not in EC (*p* = 0.0899). It should be noted, that high r values indicated strong (*r* = −0.6169) and moderate (*r* = −0.4113) effects in both EO and EC, respectively. The anticorrelated nature of the *β* ~ RVPA relationship is interesting considering that the CDE cohort was characterized by lower *β* estimates compared to the YC group. If this between-group difference signaled a dose–response-like pathological mechanism in aging that affects cognition, smaller *β* values would be associated with poorer performance metrics in the CANTAB tasks. Therefore, it appears more plausible that this reduction in the β– at least in the 2–22.5 Hz range – instead reflects a naturally occurring mechanism, where the brain is tuning E/I balance in order to better maintain cognitive performance through aging. Nevertheless, this study only identified a significant correlation between β and performance in a single task, while between-group differences in CANTAB measures were observed in four tasks in total (PAL, RTI, RVP, and SWM). Therefore, the outcomes of the present analysis do not provide support for broad claims regarding the role of cortical E/I ratio and cognition in aging and warrant further investigations.

Nevertheless, certain notions also need to be emphasized. Confluent evidence shows that the spectral slope *β* gradually reduces throughout the adult lifespan (Voytek et al., 2015; Thuwal et al., 2021), although this relationship is not necessarily linear and may exhibit a different pattern during early development in childhood (Cross et al., 2025). While an age-related decrease was strongly evident in the sample when investigated on the level of discrete age groups, age as a continuous variable was not predictive of *β*. This inconsistency may stem from multiple reasons: the sample size was relatively small, the distribution of age was not continuous, and educational background–especially of elderly participants–was homogenous and not representative of a general population. Similarly, the study observed age group-specific correlations between β and CANTAB performance metrics–most prominently for RVPA, which was significantly correlated with β in both EC and EO conditions, but only in the elderly group. However, the study sample was not suitable to consider if these relationships are continuously moderated by age, or to identify potential age thresholds at which moderation becomes significant. These considerations are important for a better understanding of the neural changes throughout aging and how they affect cognitive performance and thus call for further research, especially involving a study sample where age is continuously distributed without substantial gaps.

### 4.4 Validation of results

While IRASA is an established tool driven by theoretical considerations (i.e., the self-affinity property of scale-free signals), other available techniques are available to estimate the spectral slope β without the biasing effect of oscillatory peaks, such as the widely used FOOOF method. This study opted to utilize IRASA instead of FOOOF in the initial analysis for two main reasons. First, this study intended to remain consistent with the previous work (Czoch et al., 2024) pertaining to analytical approach. The second reason concerned spectral bimodality, where different frequency regimes are characterized by different β values (Miller et al., 2009; He et al., 2010). While FOOOF is an excellent data-driven tool to separate broadband scale-free activity from oscillations, it fits a Lorentzian model on the power spectrum and therefore only accounts for spectral bimodality where the high-frequency regime of the spectrum is steeper than the flat low-frequency regime (i.e., concave bimodality) (Donoghue et al., 2020). IRASA is not limited in this regard and instead allows for multi-modal slope assessment for both concave- and convex-type EEG spectra, as observed previously (Racz et al., 2021; Racz et al., 2024). However, initial inspection of power spectra did not indicate bimodality, so the study decided to proceed with unimodal estimation of *β* with IRASA, which was then explicitly confirmed through the bimodal MMSPM analysis. In summary, these confirmatory analyses provide additional validity to the findings, indicating that the observed changes in β can be robustly captured regardless of the choice of analytical technique. Additionally, while the present dataset imposed limitations on the applicable analytical strategies, the question of bimodality (Supplementary Figure 5) requires further investigation, particularly given prior evidence indicating altered bimodality of scale-free neural activity in healthy aging (Mukli et al., 2018).

### 4.5 Limitations and future perspectives

The presented results outline several paths forward, many of which stem from the limitations of the present study. The analyzed sample (a total of 43 participants) is too small to allow for generalizing the results to a broad population and requires validation in independent samples. Most importantly, screening for clinically relevant cognitive impairment relied on self-reporting than comprehensive neuropsychiatric evaluation. Although core elements of the Petersen criteria for MCI (Petersen et al., 2018) such as subjective memory complaints or intact activities of daily living rely primarily on self-reporting, the screening procedure in this study was not sufficient to address other facets such as preserved general cognitive functioning. While the CANTAB tasks provided quantifiable measure of cognitive functioning, they were not tailored towards rigorously identify/exclude MCI. Therefore, this sample does not allow for attributing the observed neural patterns solely to healthy aging. Validation analyses (i.e., excluding participants with SWM *z*-scores below −1.5, or subgroup analyses) indicate that even if some of the CDE participants were cognitively impaired, this likely had minimal effect on the findings. Generalizability is further limited by the fact that all CDE participants had graduate-level education, which is not representative of the general population. The EEG system employed had low spatial resolution, preventing inferences about specific brain regions and moving the focus to consider global average activity. In particular, while the administered tasks involved a visual interface and the resting-state paradigm included both EC and EO conditions, the electrode montage provided minimal coverage of the occipital cortex, limiting conclusions about visual information processing. Furthermore, built-in filtering strongly limited the available frequency regime, rendering the study sample unsuitable for multimodal slope analysis. While the correlation analysis identified a strong association between *β* and RVP performance in the elderly cohort, an EEG–cognition relationship was only confirmed for only a single task. Correlation analysis does not imply a causal relationship between E/I ratio and cognitive performance, while confirmatory analyses with higher spatial resolution and ideally employing source activity reconstruction (Michel and Brunet, 2019), are required to precisely identify the brain regions expressing altered E/I ratio and to examine their relationship to reduced cognitive performance in CDE. Such studies could provide potential intervention targets to assess if appropriately modulating E/I ratio – e.g., with transcranial direct current (Krause et al., 2013) or magnetic (Lányi et al., 2024) stimulation – could result in an improvement in sustained attention and working memory. Furthermore, this study analyzed how *β* may relate to cognitive performance in the CDE cohort but did not address how *β* or E/I ratio could explain the difference in cognitive capabilities among individuals with comparable levels of brain health (Martinez-Canada et al., 2023). This latter concept, conceptualized as *cognitive reserve* (CR) (Stern, 2003), appears fundamental for a better understanding of cognitive aging and understanding its biological determinants can be seminal for the development of novel interventions and clinical practices (Stern et al., 2023). This study did not consider CR during data collection, and thus only collected LOE from its currently established proxies. The study found a potential inverse relationship between LOE and *β* (although not significant), implying that higher education levels might predicate less steep power spectra. Furthermore, a recent systematic review indicates that no prior study investigated *β* as a potential electrophysiological signature of CR despite the confluent evidence regarding its relevance in cognition (Pereira Pereira da Silva et al., 2023). Therefore, investigating *β* as a potential EEG index of cognitive reserve appears as an important future research direction. Relatedly, LOE was significantly different between our study groups, with every participant in the CDE cohort having at least a master’s degree. This limits the generalizability of the findings and may bias between-group differences (even though LOE expressed no significant relationship with β). Furthermore, high LOE in the CDE group is especially relevant in the context of CR, as education being one of the major proxies (Nucci et al., 2012). It can be reasonably assumed that the CDE population had high CR. Therefore, the study sample in itself is unsuitable for inferring whether β is a potential signature of CR, and future research should include individuals with more diverse educational backgrounds. It is also important to note that the E/I ratio is only one hypothesis regarding the relevance of scale-free brain activity and β. 1/f -type activity is also characteristic for systems operating at – or near – criticality (Stanley, 1971), which in terms of the brain might indicate a state allowing for rapid, system-wide reorganization in response to external or internal stimuli (Beggs and Timme, 2012). There is confluent evidence pointing towards the validity of this hypothesis (Racz et al., 2018a; O'Byrne and Jerbi, 2022). Another hypothesis attributes scale-free dynamics to the interplay of antagonistic feedback mechanisms, such as those expressed by excitatory and inhibitory neuronal populations (Poil et al., 2012), effectively linking criticality to the E/I ratio. Nevertheless, throughout this manuscript, interpretation of changes in β show effect on the E/I ratio, this interpretation should be treated with caution for multiple reasons. First, there is ongoing debate whether the EEG β itself is indeed a direct proxy of the E/I ratio (Cohen Kadosh, 2025; Chakravarty et al., 2025). The limited frequency regime available in the sample (2–22.5 Hz) also restricted the ability to address potential discrepancies in the slope–E/I ratio relationship between low- and high-frequency regimes (Becker et al., 2018; Muthukumaraswamy and Liley, 2018). Inference on the relationship between the β and the E/I ratio should also be supported by experimental modulation of the E/I ratio, such as transcranial magnetic (Lányi et al., 2024) or electrical (Kang et al., 2025) stimulation, or other interventions (e.g., pharmaceutical). Such considerations need to be addressed in future research in order to truly link the observed EEG patterns in CDE to the changes in the E/I ratio, and potentially establish a causal relationship between neurobiology and cognitive performance. Finally, this study mainly focused on the spectral slope β, it is not the only relevant electrophysiological marker and potential neural signature in cognitive aging (Tanaka et al., 2024). More comprehensive approaches are needed in characterizing neural signatures in aging.

## 5 Conclusion

This study contributes to the growing evidence indicating a reduced β in healthy aging and its association with cognitive performance. This study found a strong, anticorrelated relationship between β and performance in a rapid visual information processing task, though not across the tasks, which could only be confirmed in the elderly population when resting with eyes open. This pattern taken together with the between-group difference suggests that the flattening of the power spectrum might point to a compensatory mechanism of the brain to account for deteriorating cognition naturally occurring with aging. Further studies are required to link this mechanism to specific brain regions, paving the way for the development for novel intervention strategies aiming for appropriately modulating cortical E/I ratio in order to preserve, improve, or even restore cognitive capabilities in the elderly.

## Data Availability

The raw data supporting the conclusions of this article will be made available by the authors, without undue reservation.
