# Supplementary material for: Global scale-free brain activity as a potential neural signature of visual information processing in aging
Source: Front Aging Neurosci. 2026 Apr 1;18:1770204. doi: 10.3389/fnagi.2026.1770204 (PMC13080313; doi:10.3389/fnagi.2026.1770204)
Supplement: Supplementary file 1 [file Data_Sheet_1.docx]

Supplementary Material - Global scale-free brain activity is associated with visual information processing in healthy aging

Frigyes Samuel Racz, Zalan Kaposzta, Akos Czoch, Joshua T. Chang, Orestis Stylianou, Peter Mukli, Jared F. Benge and Andras Eke

# Complete list of CANTAB performance measures

A complete exploratory analysis of all 154 CANTAB measures is provided in tabular format as a separate Supplementary File (see Supplementary File 1). This table contains the following information:

- **Name**: Abbreviated name of CANTAB output measure. For full more details on each CANTAB task and output measures, please see <https://cambridgecognition.com/digital-cognitive-assessments/>, specifically:
  - MOT: <https://cambridgecognition.com/motor-screening-task-mot/>
  - DMS: <https://cambridgecognition.com/delayed-matching-to-sample-dms/>
  - PAL: <https://cambridgecognition.com/paired-associates-learning-pal/>
  - PRM: <https://cambridgecognition.com/pattern-recognition-memory-prm/>
  - RTI: <https://cambridgecognition.com/reaction-time-rti/>
  - RVP: <https://cambridgecognition.com/rapid-visual-information-processing-rvp/>
  - SWM: <https://cambridgecognition.com/spatial-working-memory-swm/>
- **Young**: Group expected value and spread in the young cohort. In case the data was normally distributed (as assessed via Lilliefors test), data is presented in the format ‘mean (standard deviation)’, while as ‘median [inter-quartile rage]’ otherwise.
- **Elderly**: Group expected value and spread in the elderly cohort, presented in similar manner as described for Young.
- **Test**: Statistical test used for contrasting CANTAB metrics between young and elderly cohorts (two-sample t test or Mann-Whitney U test, depending on data normality).
- **Statistic**: The value of the test statistic (*t* or *z*) for the given comparison.
- **p**: *p*-value.
- **alpha**: Level for statistical significance as adjusted using the False Discovery Rate technique.
- **H**: Outcome of the hypothesis test.
- **ES**: Effect size as captured via Cohen’s *d* for normally distributed data and rank-biserial correlation (*r*) otherwise.

# Correlation between RVPA and Spectral Slope β in the elderly cohort


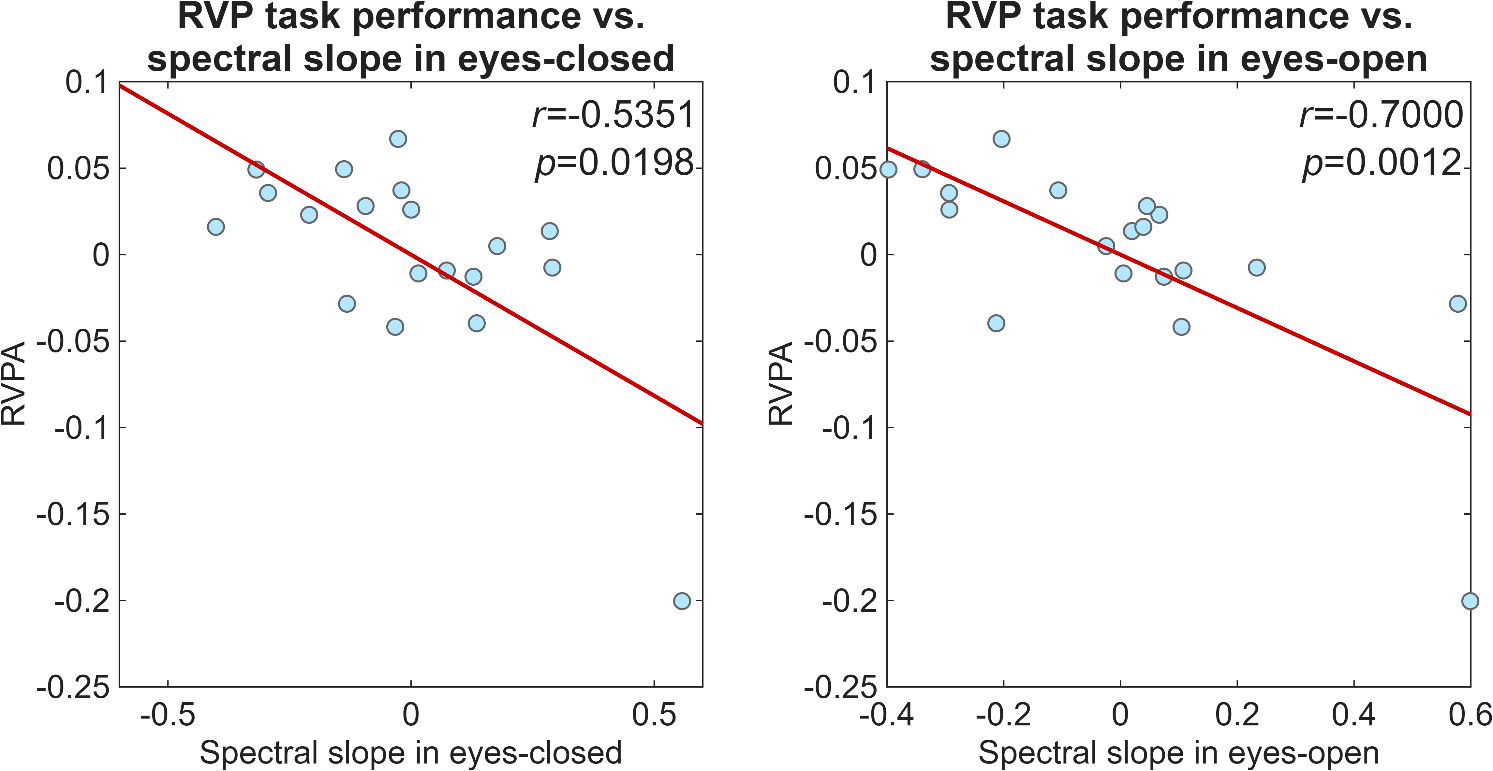


Supplementary Figure 1. Correlation between RVPA and EEG spectral slope in eyes-closed (left) and eyes-open (right) conditions in the community-dwelling elderly group. Figure shows analysis of the complete elderly cohort, including one outlier (bottom right) with RVPA score 3.5 standard deviations below the group average (after removing confounding variables). Spearman correlation coefficient *r* and the corresponding *p*-value are presented on both panels. The thick red line indicates the least-squares fit.

# Channel-wise Analyses


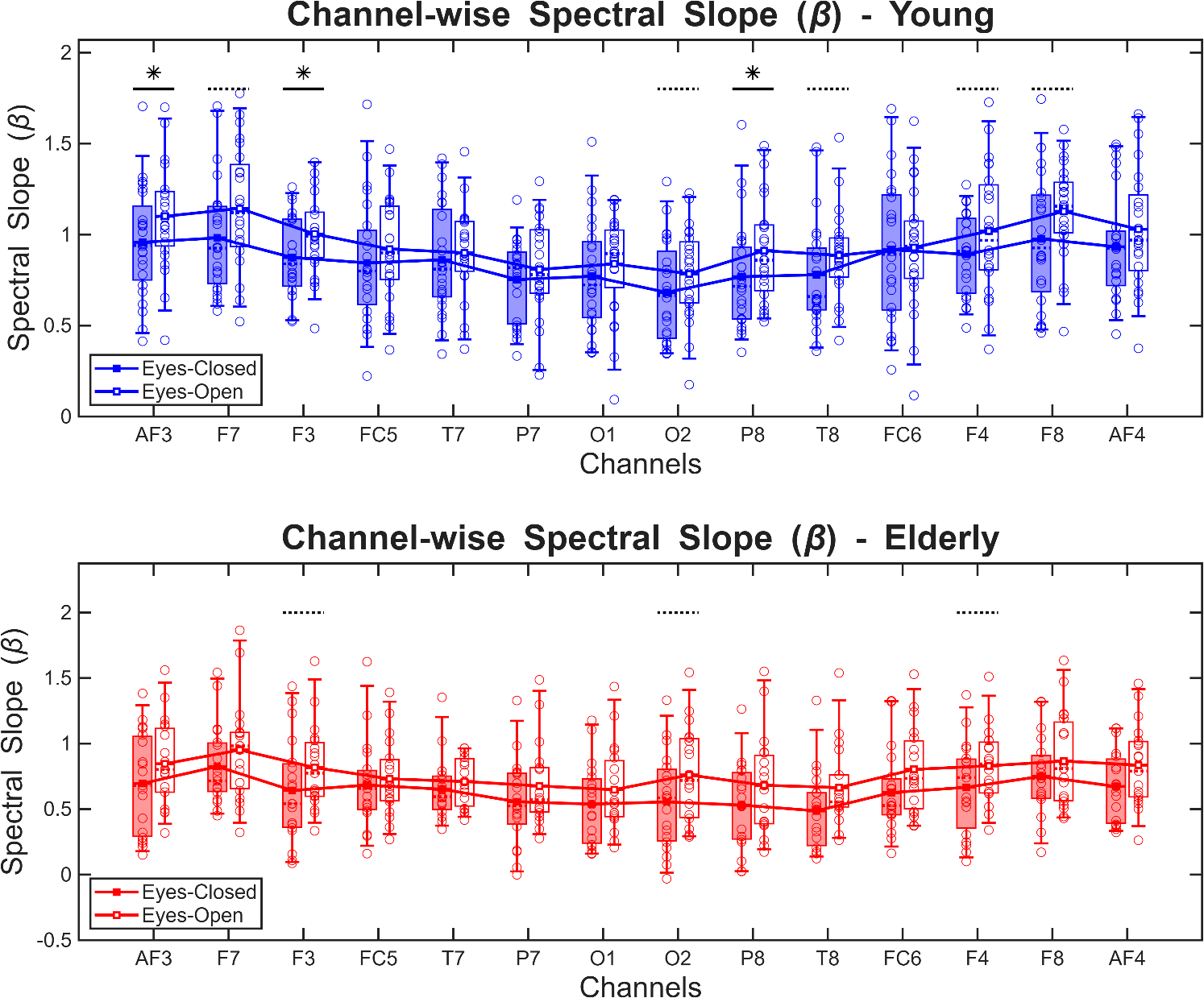


Supplementary Figure 2. Channel-wise EEG spectral slopes in eyes-closed and eyes-open states, in the young (upper) and elderly (lower) cohorts. Thick horizontal lines indicate significant between-group differences, while dotted line denote between-group differences rendered non-significant by multiple comparisons adjustment. * indicates *p*<0.05.


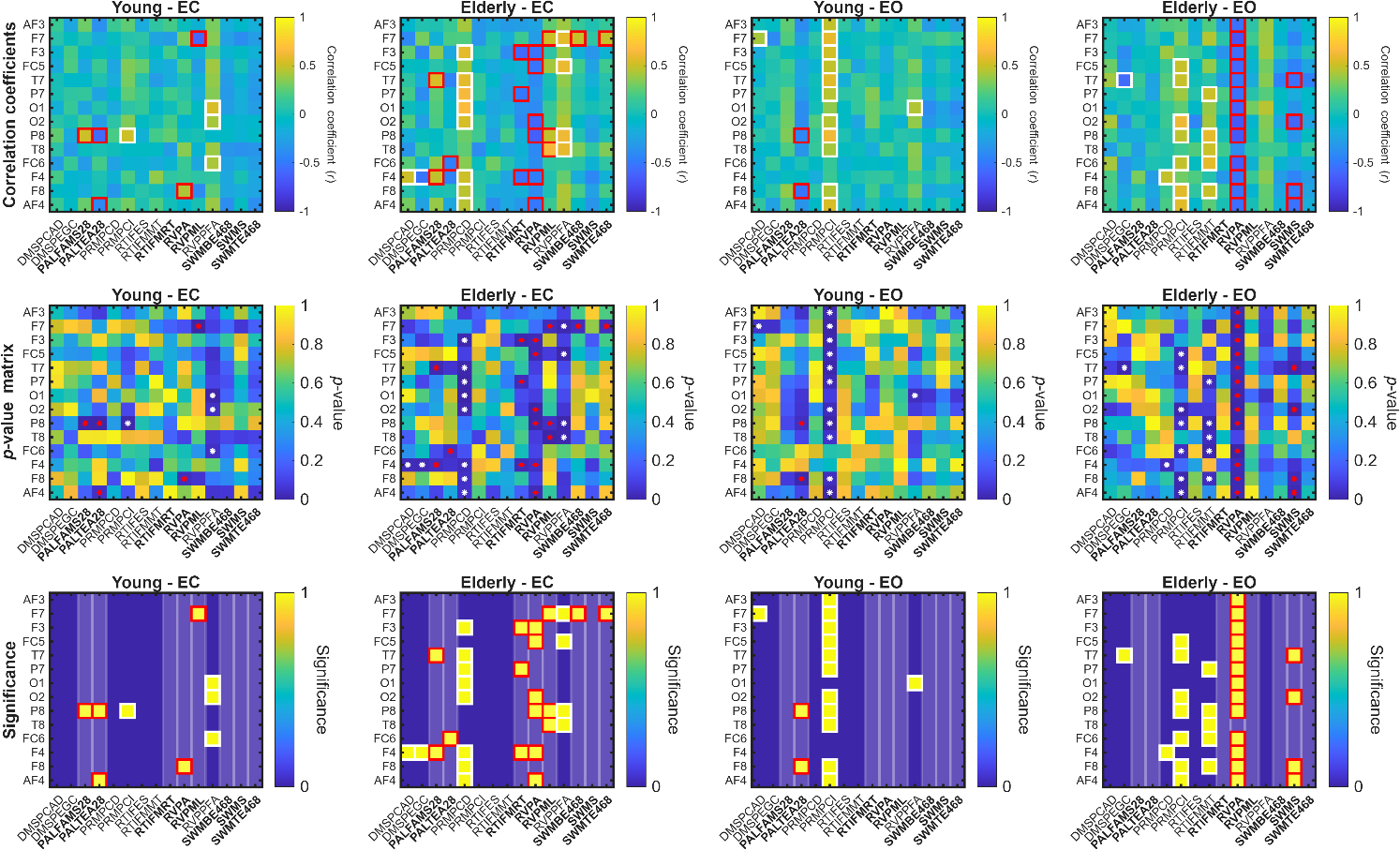


Supplementary Figure 3. Complete correlation analysis between channel-wise spectral slope β and CANTAB output measures. Columns show outcomes from the four age group-physiological state combinations from left to right as young with eyes-closed (Young – EC), elderly with eyes-closed (Elderly – EC), young with eyes-open (Young – EO) and elderly with eyes-open (Elderly – EO). In each matrix, columns denote the 15 CANTAB test scores, while rows denote the 14 EEG scalp locations. Matrices in the top row show the Pearson- or Spearman correlation coefficient (*r*) values depending on data normality, while those in the middle and bottom rows present the corresponding *p*-values and indicated statistical significance (*p*<0.05), respectively. In all panels, those CANTAB measures that were significantly different between young and elderly groups are denoted in bold and marked with an asterisk. This is also further illustrated in the bottom panel by slightly lighter shading. Those β-CANTAB correlations that were statistically significant (*p*<0.05) are indicated by thick frames in the top and bottom panels, while asterisk symbols in the middle panel. Finally, on all panels, significant correlations for CANTAB measures that indicated a performance difference between young and elderly cohorts are denoted with red color, while the rest is marked in white.

# Validation Analyses

## MMSPM analysis

Global power spectra were obtained as described in the main text. Specifically, the obtained raw power spectra from IRASA analysis were used as input to the MMSPM algorithm. The MMSPM algorithm was utilized with the following parameter settings:

- s_env = [1,2,4,8,16,32]
- f_bp = 'auto'
- f_ma = 2
- f_w = 0.5
- q_lo = 0.4
- max_peak_N = 3
- min_peak_Amp = 0.25
- min_peak_SD = 1.5
- max_width = 8
- th_peak_heigh = 0.1
- th_peak_width = 2
- th_edge = 1
- th_exclude = 2
- flag_fig = false
- flag_disp = false
- debug = false

For details on the specific functions of these parameters, please see the online documentation for MMSPM at <https://github.com/samuelracz/MMSPM>. Unimodal spectral slopes were extracted and analyzed using the same pipeline as those obtained via IRASA in the original analyses. The MMSPM method with unimodal and bimodal settings is illustrated on **Supplementary Figure 4** and **Supplementary Figure 5**, respectively.


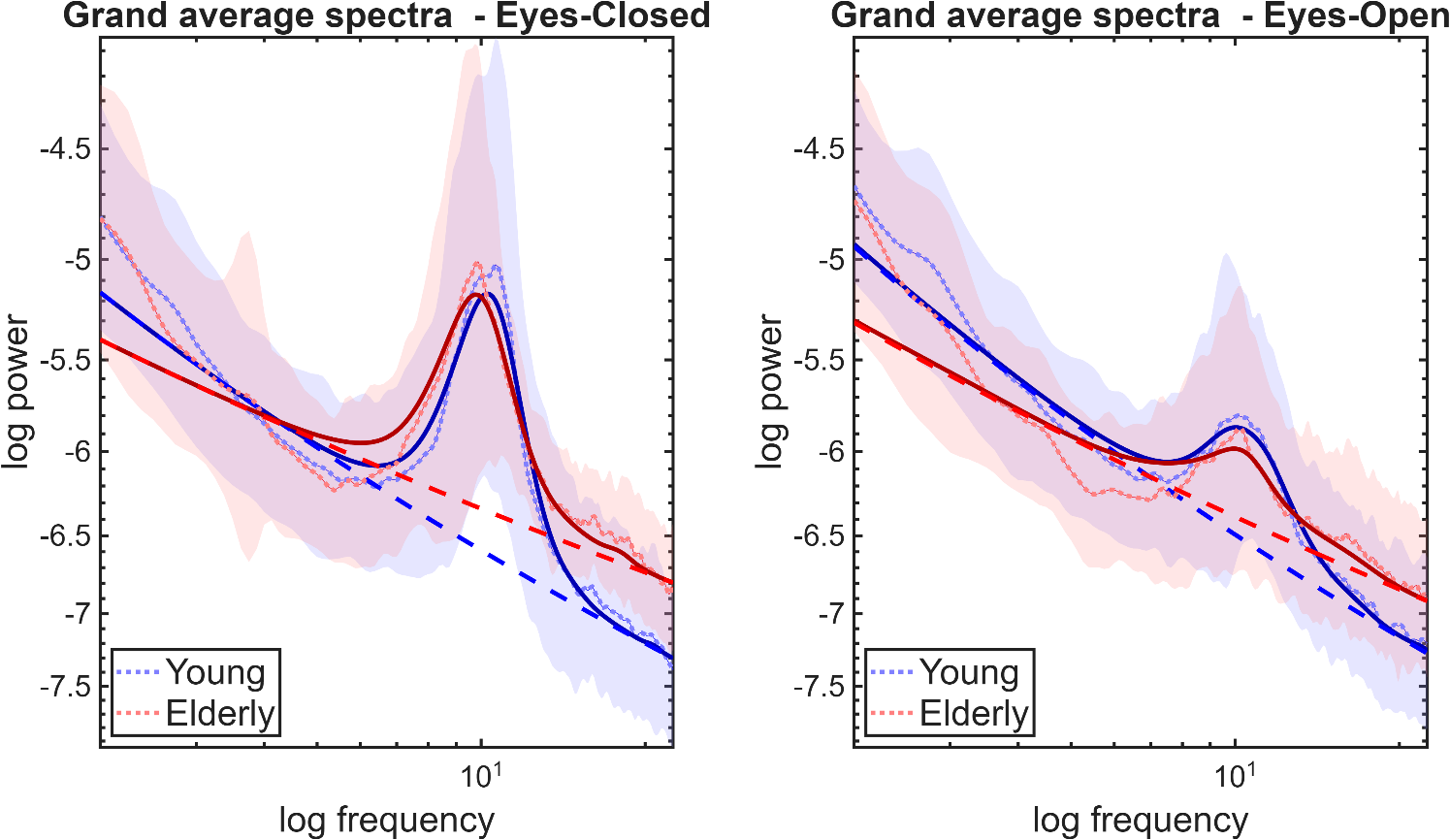


Supplementary Figure 4. Separating broadband fractal and oscillatory components in the power spectrum using MMSPM with *unimodal* settings. Dotted and thick lines denote the raw power spectrum and the isolated fractal component, respectively. The shaded areas indicate the standard deviation as observed in raw power spectra, while the dashed lines illustrate the power-law fit obtained from the fractal component.


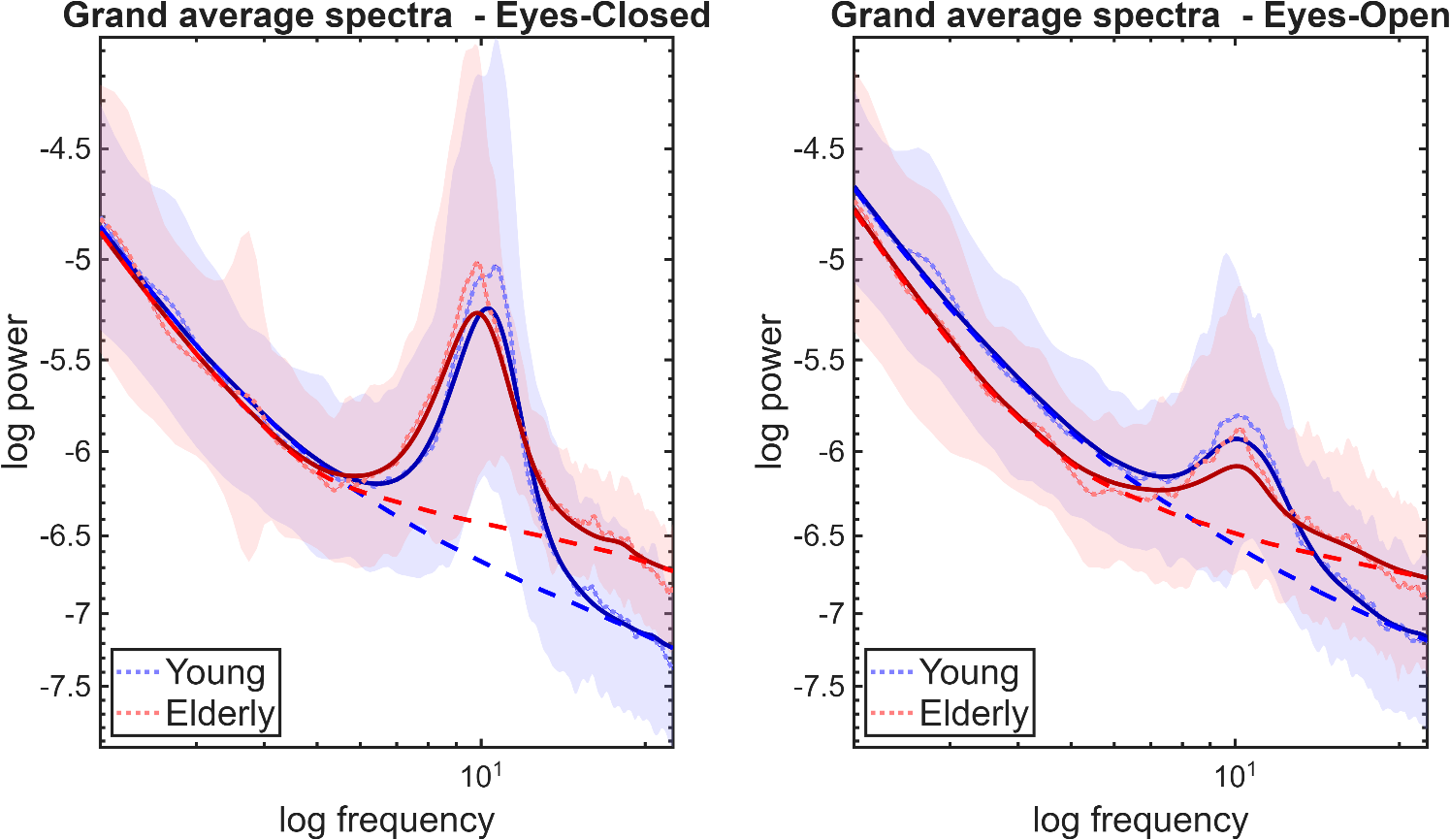


Supplementary Figure 5. Separating broadband fractal and oscillatory components in the power spectrum using MMSPM with *bimodal* settings. Dotted and thick lines denote the raw power spectrum and the isolated fractal component, respectively. The shaded areas indicate the standard deviation as observed in raw power spectra, while the dashed lines illustrate the power-law fit obtained from the fractal component.

## Addressing Spectral Bimodality

While grand average power spectra suggested concave-type bimodality on the group level, this could not be robustly confirmed on the individual level. Median breakpoint positions and the number of participants with significant bimodality (*N_bimodal_*) are reported in **Supplementary Table 1**. As less than 50% of participants exhibited statistically significant bimodality in each analysis subgroups, we proceeded with unimodal analysis.

Supplementary Table 1. Exploring spectral bimodality in study cohorts.

| **Group** | **State** | **Median Breakpoint** | ***N_bimodal_*** |
| --- | --- | --- | --- |
| Young | Eyes Closed | 6.2670 Hz | 10 out of 24 |
|  | Eyes Open | 7.5625 Hz | 4 out of 24 |
| Elderly | Eyes Closed | 5.1905 Hz | 9 out of 19 |
|  | Eyes Open | 5.4163 Hz | 9 out of 19 |

## Effect of Age and Physiological State on Global Spectral Slope – Results from Unimodal MMSPM Analysis

We could not utilize the formerly employed parametric statistical pipeline as EO slope estimates from the CDE cohort was not normally distributed (Lilliefors test, $p=0.0118$). Instead, pairwise comparisons were performed. Between-group analyses revealed decreased β in HE compared to HY on both EC (two sample t test, HY: $0.8837\pm0.2524$ vs. HE: $0.5697\pm0.2659$, $t_{41}=3.9574$, $p=0.0003$) and EO (Mann-Whitney U test, YC: $0.9629, IQR: [0.8817;1.1153]$ vs. CDE: $0.5664, IQR:[0.5032;0.7861]$, $z=3.4850$, $p=0.0005$) states, while post-hoc within-group analysis revealed an significant increase in β in the YC (paired t test, $t_{23}=2.2128$, $p=0.0371$) but not in the CDE group, as for the latter the difference was not significant (Wilcoxon signed rank test, z$=1.1670$, $p=0.2432$). These results are illustrated in **Supplementary Figure 6**.


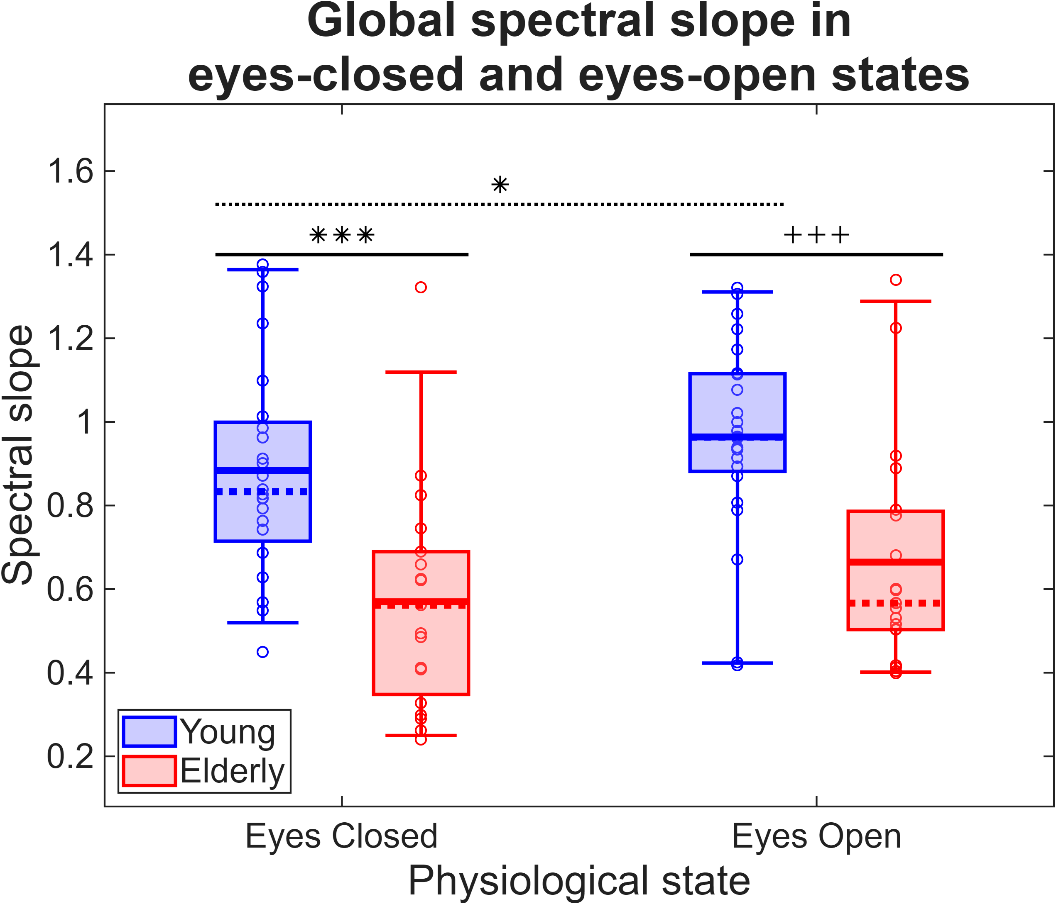


Supplementary Figure 6. EEG spectral slopes in young and elderly cohorts, as obtained via unimodal MMSPM analysis. Thick horizontal lines indicate significant between-group differences, while dotted line denotes significant within-group difference. Asterisk (*) and plus (+) symbols denote parametric and non-parametric statistical testing, respectively. One, two and three symbols indicate *p*<0.05, *p*<0.01 and *p*<0.001, respectively.

## Association Between Spectral Slope and Cognitive Performance – Results from Unimodal MMSPM Analysis

Similarly to the IRASA pipeline, correlation analysis only revealed a significant relationship between spectral slope and RVPA in the elderly group (**Supplementary Figure 7**). The Pearson correlation coefficient was significant in the EO state (*r*=-0.6632, *p*=0.0027), while marginally significant in EC (*r*=-0.4431, *p*=0.0656).


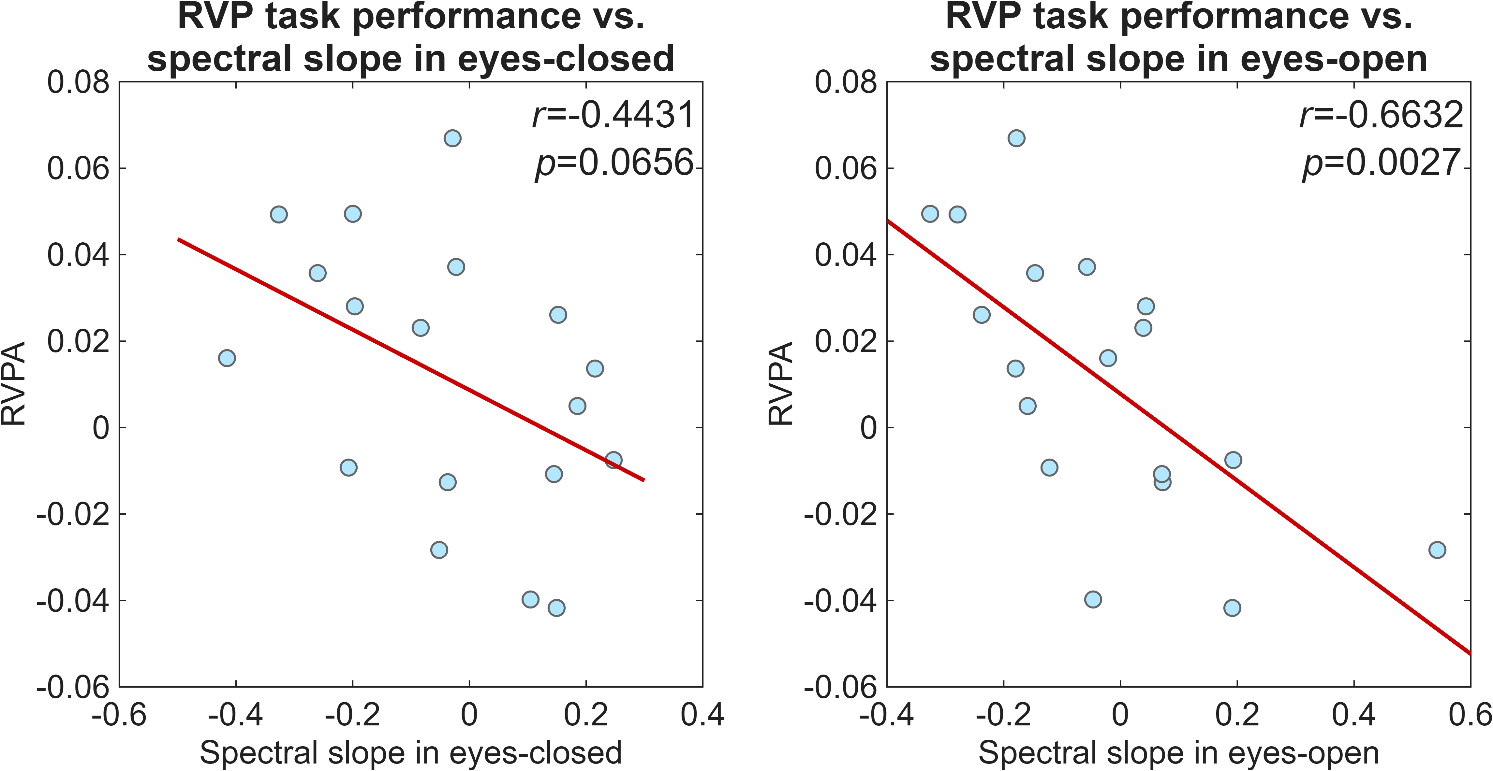


Supplementary Figure 7. Correlation between RVPA and EEG spectral slope – as obtained via unimodal MMSPM analysis – in eyes-closed (left) and eyes-open (right) conditions. Figure shows analysis of data after one outlier has been removed, whose RVPA score was 3.5 standard deviation below the group mean. The thick red line indicates the least-squares fit.

## Sensitivity analysis in the elderly group

Here we assessed the reproducibility of results after excluding elderly participants with $\boldsymbol{z<-1.5}$ in PAL and SWM tasks. On the reduced CDE sample (CDE-R, $n=17$), the repeated measures ANOVA indicated a significant main effect of group ($F=10.23$, $p=0.0027$, $\eta_{p}^{2}=0.2078$) and physiological state ($F=8.0254$, $p=0.0073$, $\eta_{p}^{2}=0.2707$), however no significant age group $\times$ physiological state interaction was found ($F=0.0535$, $p=0.8183$, $\eta_{p}^{2}=0.0014$). Post-hoc between-group analysis revealed decreased β in CDE-R compared to YC on both EC (YC: $0.8604\pm0.2349$ vs. CDE-R: $0.6350\pm0.2676$, $t_{39}=2.8581$, $p=0.0068$, $d=0.8885$) and EO (YC: $0.9624\pm0.2101$ vs. CDE-R: $0.7551\pm0.2868$, $t_{39}=2.6749$, $p=0.0109$, $d=0.8315$) states, while post-hoc within-group analysis revealed a significant increase in β in the YC ($t_{23}=2.9613$, $p=0.0070$, $d=0.5845$) but not in the CDE-R group ($t_{16}=1.5058$, $p=0.1516$, $d=0.3478$). These results are illustrated in **Supplementary Figure 8**.

**
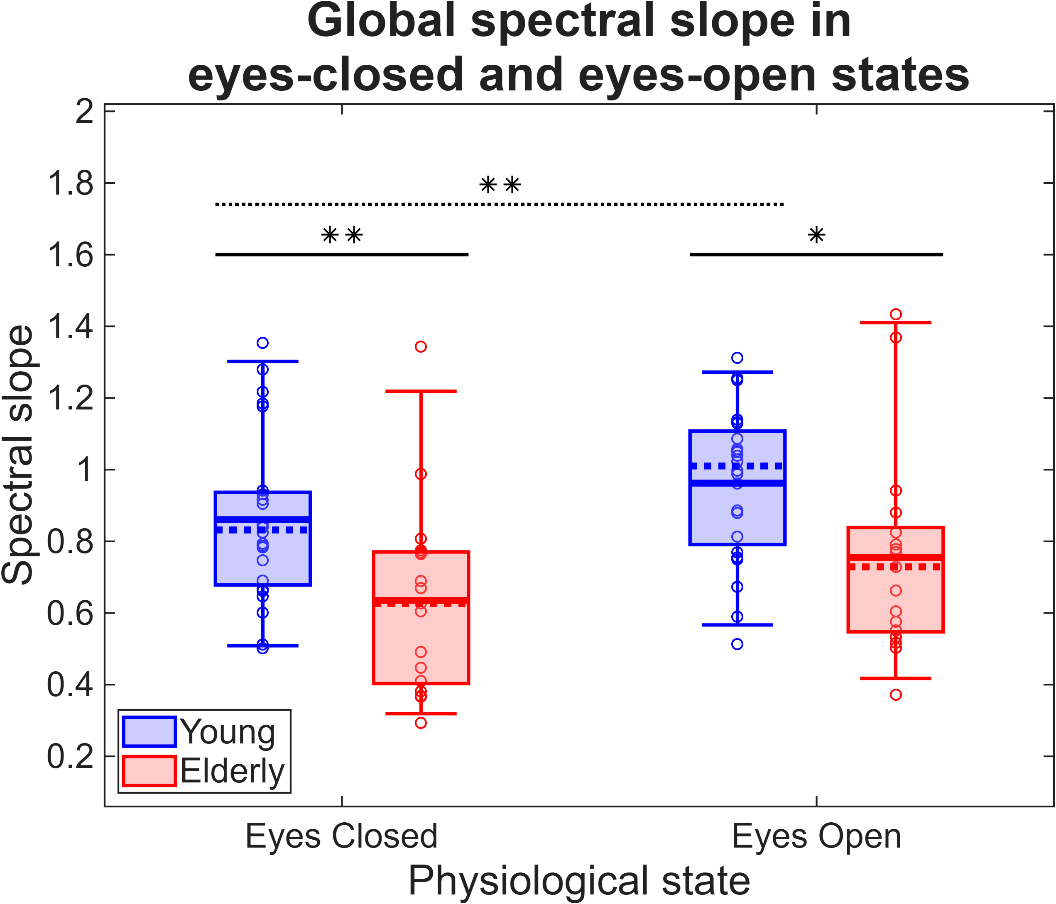
**

Supplementary Figure 8. EEG spectral slopes in young and elderly cohorts after excluding elderly participants with $\boldsymbol{z<-1.5}$ in the PAL or SWM tasks. Thick horizontal lines indicate significant between-group differences, while dotted line denotes significant within-group difference. * and ** indicate *p*<0.05 and *p*<0.01, respectively.

The complete correlation analysis is illustrated on **Supplementary Figure 9**, which is in complete alignment with the outcomes from the full cohort, shown on **Figure 4** in the main text. The singled-out Spearman correlation coefficient for the RVPA-β relationship was significant both in the EO (*r*=-0.7404, *p*=0.0007) and EC (*r*=-0.6893, *p*=0.0022) states (**Supplementary Figure 10**), including an outlier subject. Exclusion of this participant rendered the data normally distributed with Pearson correlation analysis indicating the relationship with β significant in the EO state (*r*=-0.5941, *p*=0.0152), but not in EC (*r*=-0.3699, *p*=0.1585).


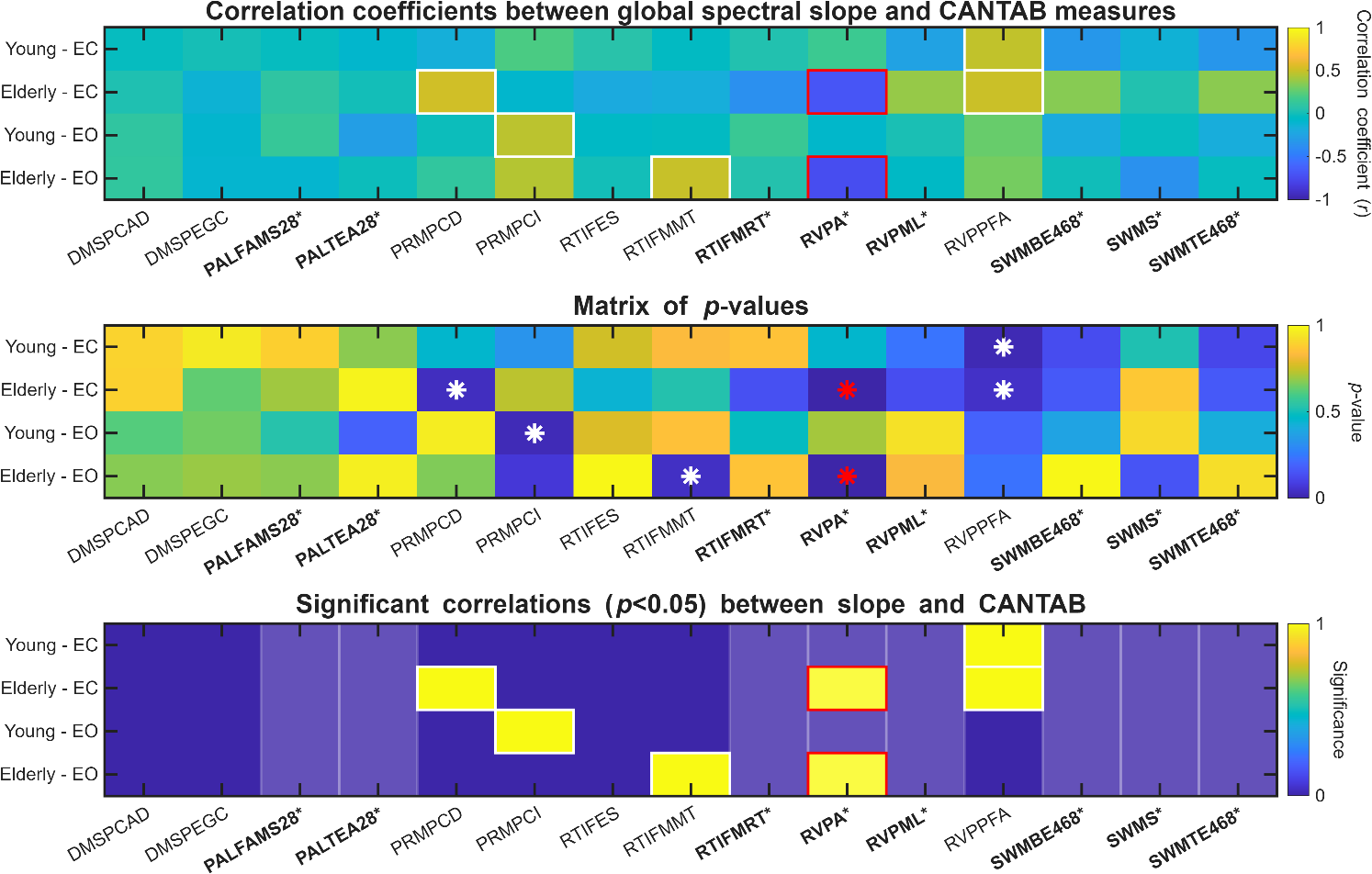


Supplementary Figure 9. Complete correlation analysis between spectral slope β and CANTAB output measures after excluding elderly participants with $\boldsymbol{z<-1.5}$ in the PAL or SWM tasks. The top matrix shows the Pearson- or Spearman correlation coefficient (*r*) values depending on data normality, while the middle and bottom panels present the corresponding *p*-values and indicated statistical significance (*p*<0.05), respectively. In all panels, those CANTAB measures that were significantly different between young and elderly groups are denoted in bold and marked with an asterisk. This is also further illustrated in the bottom panel by slightly lighter shading. Those β-CANTAB correlations that were statistically significant (*p*<0.05) are indicated by thick frames in the top and bottom panels, while asterisk symbols in the middle panel. Finally, on all panels, significant correlations for CANTAB measures that indicated a performance difference between young and elderly cohorts are denoted with red color, while the rest is marked in white.


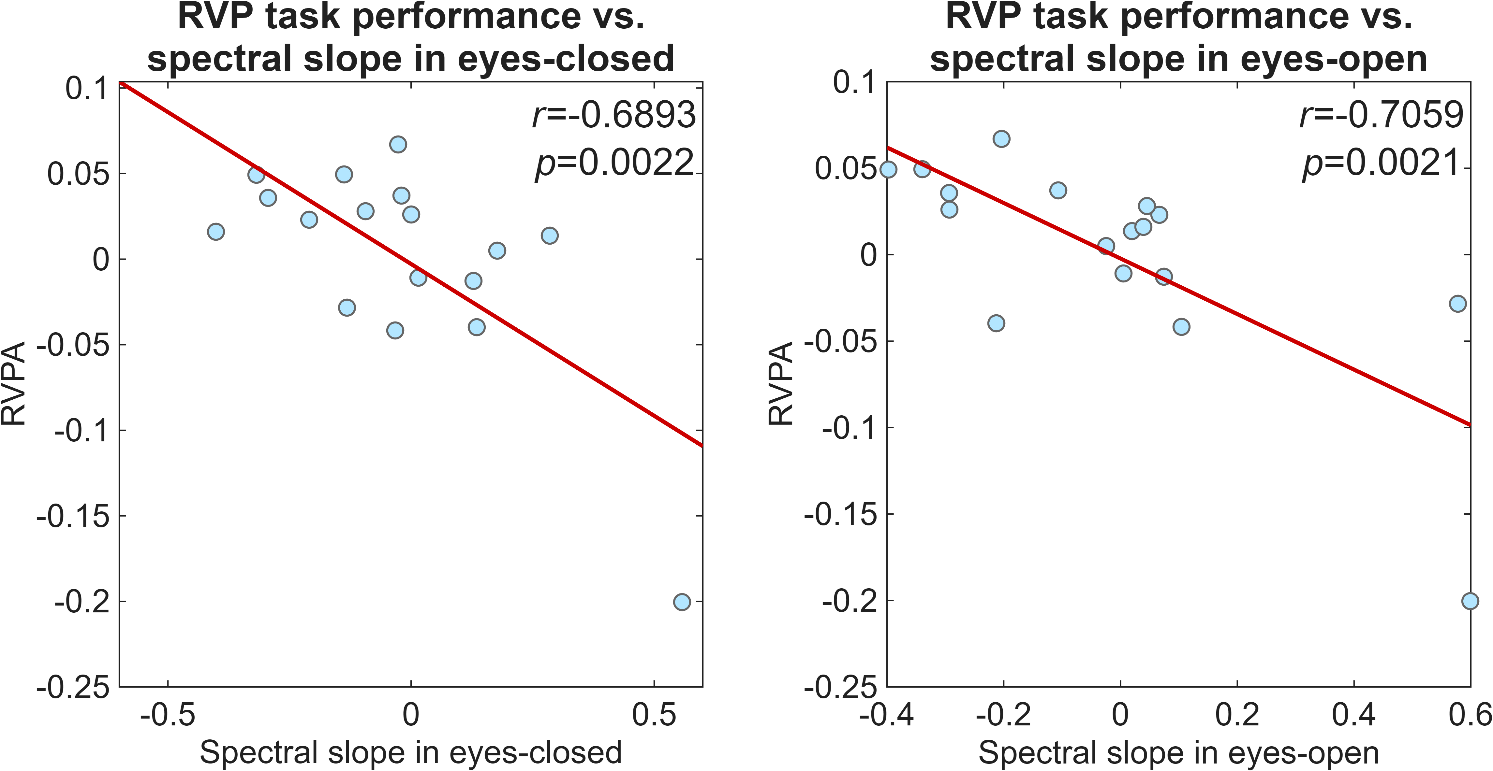


Supplementary Figure 10. Correlation between RVPA and EEG spectral slope in eyes-closed (left) and eyes-open (right) conditions in the elderly group after excluding two participants with $\boldsymbol{z<-1.5}$ in the PAL or SWM tasks. Figure shows analysis including one outlier (top left) with RVPA score 3.5 standard deviations below the group average (after removing confounding variables). Spearman correlation coefficient *r* and the corresponding *p*-value are presented on both panels. The thick red line illustrates the least-squares fit.


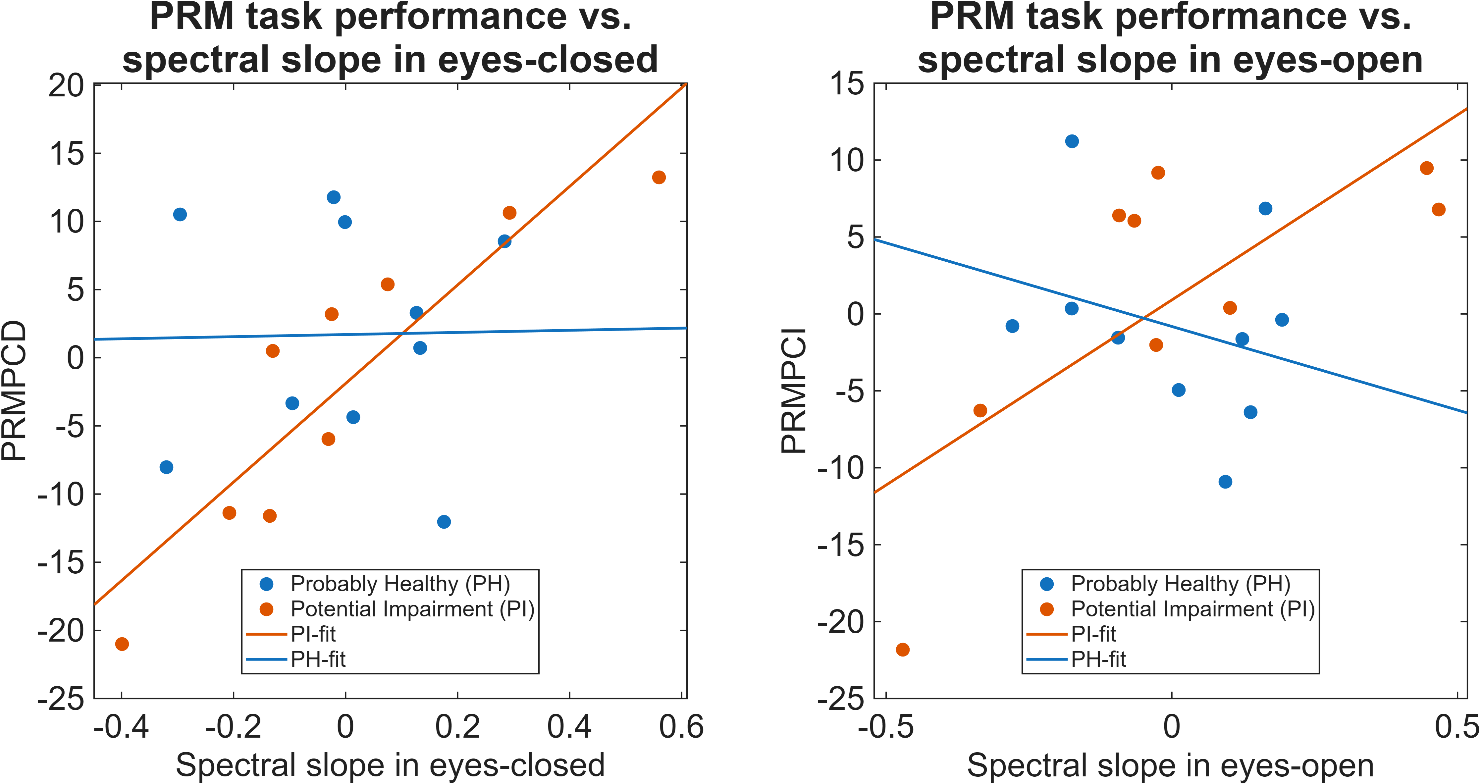


Supplementary Figure 11. Correlation between PRMPCD (left) and PRMPCI (right) and EEG spectral slope in eyes-closed (left) and eyes-open (right) conditions in the elderly group with probably healthy (PH, blue) and potential impairment (PI, orange) subgroups.
